# Efficient enzymatic synthesis and dual-colour fluorescent labelling of DNA probes using long chain azido-dUTP and BCN dyes

**DOI:** 10.1093/nar/gkw028

**Published:** 2016-01-26

**Authors:** Xiaomei Ren, Afaf H. El-Sagheer, Tom Brown

**Affiliations:** 1 Department of Chemistry, University of Oxford, Chemistry Research Laboratory, 12 Mansfield Road, Oxford OX1 3TA, UK; 2 Chemistry Branch, Department of Science and Mathematics, Faculty of Petroleum and Mining Engineering, Suez University, Suez 43721, Egypt

## Abstract

A sterically undemanding azide analogue of dTTP (AHP dUTP) with an alkyl chain and ethynyl attachment to the nucleobase was designed and incorporated into DNA by primer extension, reverse transcription and polymerase chain reaction (PCR). An azide-modified 523 bp PCR amplicon with all 335 thymidines replaced by AHP dU was shown to be a perfect copy of the template from which it was amplified. Replacement of thymidine with AHP dU increases duplex stability, accounting in part for the high incorporation efficiency of the azide-modified triphosphate. Single-stranded azide-labelled DNA was conveniently prepared from PCR products by λ-exonuclease digestion and streptavidin magnetic bead isolation. Efficient fluorescent labelling of single and double-stranded DNA was carried out using dyes functionalized with bicyclo[6.1.0]non-4-yne (BCN) via the strain-promoted alkyne-azide cycloaddition (SPAAC) reaction. This revealed that the degree of labelling must be carefully controlled to achieve optimum fluorescence and avoid fluorescence quenching. Dual-coloured probes were obtained in a single tube fluorescent labelling reaction; and varying the ratios of the two dyes provides a simple method to prepare DNA probes with unique fluorescent signatures. AHP dUTP is a versatile clickable nucleotide with potentially wide applications in biology and nanotechnology including single molecule studies and synthesis of modified aptamer libraries via SELEX.

## INTRODUCTION

Probe-based fluorescence detection is an important tool for imaging and detection of specific DNA and RNA sequences in polymerase chain reaction (PCR) amplicons, fixed cells and tissues (1–3). However, in applications where the target nucleic acid concentration is low, weak signals are obtained and detection is sometimes unsuccessful. The introduction of a high density of fluorophores into DNA probes provides an obvious solution to this problem (4,5). To achieve this, multiple fluorescently-labelled nucleotides have been incorporated directly into DNA probes during PCR or nick-translation using polymerase enzymes (6,7). However, this direct enzyme-mediated incorporation strategy is inefficient due to the bulky nature of fluorescent dyes, which are poorly compatible with DNA polymerases. Alternative post-labelling strategies have been developed whereby reactive groups are introduced into the DNA probe via a modified triphosphate and serve as substrates for subsequent fluorescent labelling (8,9). The advantage of this indirect method is that the reactive groups are sterically relatively undemanding, so the modified nucleotides are incorporated efficiently by polymerase enzymes.

Traditionally the most common post-labelling chemistry has involved coupling dye succinimidyl esters to amino-modified DNA (10,11). More recently the highly efficient copper catalysed alkyne-azide cycloaddition reaction (CuAAC) (12–16), strain-promoted alkyne-azide cycloaddition reaction (SPAAC) (17–21), Diels-Alder (DA) and inverse electron demand Diels-Alder (IEDDA) reaction (19,22–24) have been used. Of these reactions, the SPAAC reaction between cyclooctyne and azide is a good choice because it is fast, orthogonal to the functional groups in DNA, it does not require metal catalysts (which can lead to DNA degradation if not carried out with care) and it involves the non-bulky azide group. With the SPAAC reaction in mind various cycloalkyne-modified dNTPs have been incorporated into DNA using different polymerase enzymes followed by labelling with fluorophore azides (18,21). However, the bulky nature of cyclooctyne moiety makes these modified dNTPs poor substrates for DNA and RNA polymerases. For this reason the introduction of sterically undemanding azide triphosphates into DNA is a more logical strategy.

To this end, a number of azide-labelled nucleoside triphosphates have been attached to the 3′-terminus of RNA and DNA strands using nucleotidyl transferase enzymes (20,21,25). However, the method is of limited scope as only a few consecutive azide modifications can be added, and internal sequence modification is not possible. Azidoalkyl-modified uridine triphosphate has been incorporated into RNA throughout its sequence via transcription (26,27), and DNA functionalization has been achieved via primer extension using azide-modified triphosphates followed by Staudinger or CuAAC post-labelling (28,29). However, the requirement for anaerobic labelling condition and the moderate enzymatic incorporation efficiency of these particular modified nucleotides has limited the implementation of these strategies. Taking these points into account, provided that a suitable azide-modified dNTP can be designed, the SPAAC reaction should be an excellent tool for the fluorescent labelling of DNA by polymerase-based methods, as it should not suffer from the drawbacks of other click reactions.

**Figure 1.**
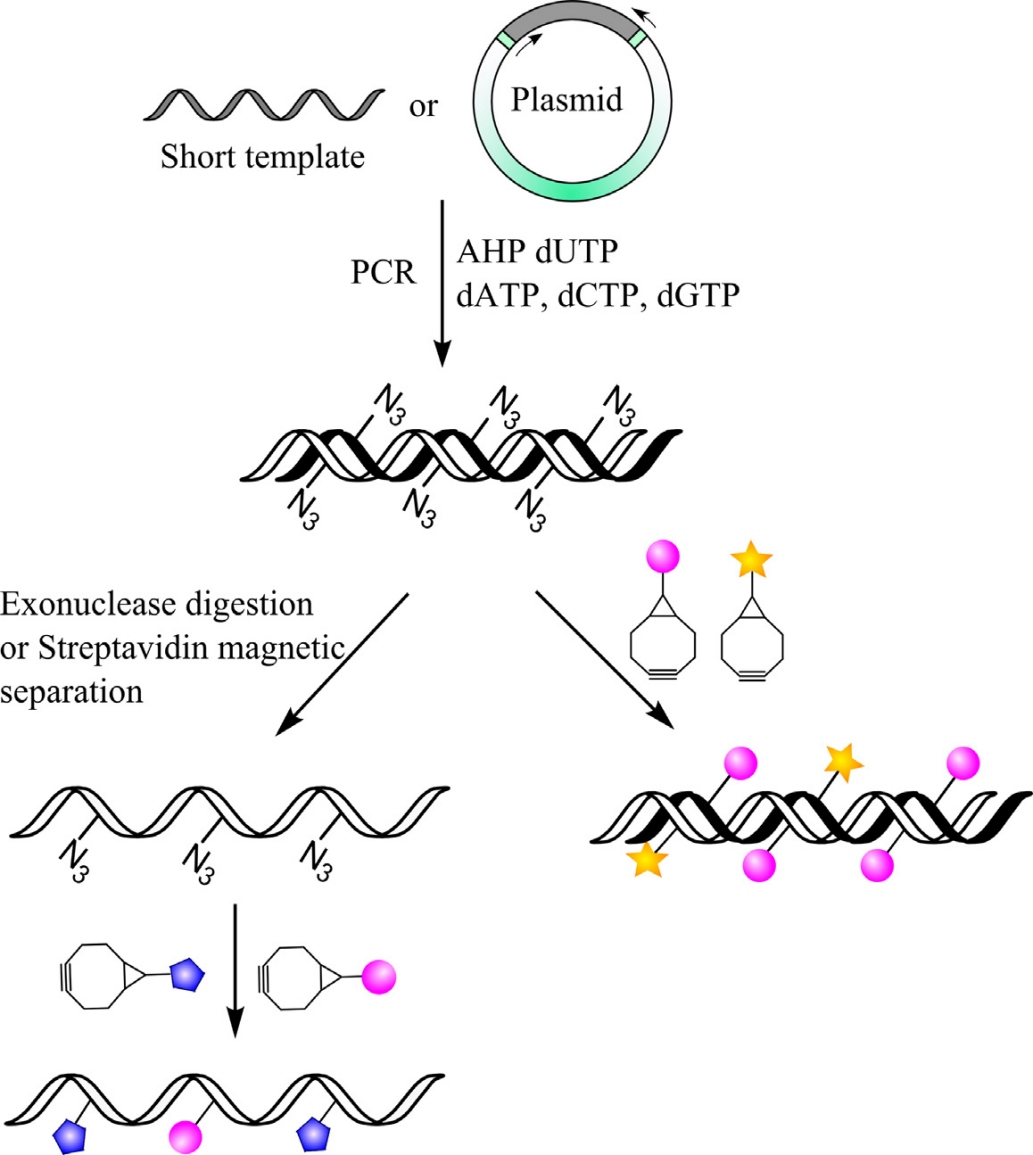
General scheme for fluorescent labelling of AHP-modified single and double-stranded DNA via the SPAAC reaction. The two curved arrows on the plasmid indicate the amplification direction and the grey section is the template region.

Our previous work on azidomethyl dUTP (AM dUTP) demonstrated that DNA polymerases can incorporate this nucleotide into DNA during primer extension (linear copying), PCR and reverse transcription (19). However, the azidomethyl modification destabilizes the DNA duplex, adversely affecting its incorporation efficiency, and the short linker between the azide and nucleobase can result in inefficient labelling due to steric hindrance from the DNA chain. To overcome these problems, a new 6-azidohexanamidopropargyl deoxyuridine triphosphate (AHP dUTP) is now reported, which has a triple bond at the 5-position of the nucleobase for higher duplex thermal stability, hence more efficient incorporation by polymerase enzymes, and a longer azidoalkyl linker to minimize steric hindrance during fluorescent labelling. The enzymatically synthesized azide-labelled DNA products can be efficiently post-labelled with fluorophores functionalized with bicyclo[6.1.0]non-4-yne (BCN) via the SPAAC reaction to produce densely labelled fluorescent single- and double-stranded DNA probes (Figure 1). A labelling strategy has also been developed to prepare dual-coloured fluorescent probes using a mixture of two different BCN-fluorophores. This methodology can be used to synthesize cocktails of DNA probes with subtly different fluorescence characteristics and could be extended to multi-coloured constructs.

## MATERIALS AND METHODS

### Materials

All chemical reagents were purchased from Sigma-Aldrich, Alfa Aesar, Acros Organics, SynAffix or Fisher Scientific. Unmodified nucleoside triphosphates were purchased from Sigma-Aldrich or Roche Diagnostics GmbH. KOD DNA polymerase was purchased from Merck Millipore and Gotaq was obtained from Promega. Klenow large fragment, Therminator™ II, M-MuLV (RNase H^−^) reverse transcriptase, AMV reverse transcriptase, RNase inhibitor and λ-exonuclease were purchased from New England Biolabs. Plasmid HydGdCTD5 (5.176 kb,PCR template) was provided by Professor Peter Roach at Southampton University School of Chemistry.

### Methods

Protocols for the synthesis of nucleoside triphosphates, BCN-modified fluorophores, oligonucleotides, primer extension reactions, kinetics measurements, fluorescent labelling of primer extension products, reverse transcription, PCR and exonuclease digestion are described in the Supplementary Methods.

### Fluorescent labelling of double-stranded DNA probes

AHP-modified or unmodified PCR amplicons from Template T11 (supporting information S6) were purified by agarose gel electrophoresis using a Qiagen gel extraction kit. The amplicons were quantified using a NanoDrop 2000 UV spectrometer. A total of 1.2 μg aliquots of T11 amplicons were diluted with Gotaq green buffer solution (60 μl, 1×) and labelled with 10 nmol of BCN-functionalized fluorophores (either a single fluorophore or a mixture of two different fluorophores, 2 mM stock solution of each dye in DMSO) at RT for 30 min. Samples were then analysed on a 1.5% agarose gel containing ethidium bromide (200 ng/ml) at a constant voltage (126 V) in 1× Tris-Borate-EDTA (TBE) buffer. Labelled products were extracted from the agarose gel and aqueous solutions (50 μl) were obtained. The extracted solutions were diluted with deionized water (150 μl) or 100 mM TEAB buffer (150 μl, pH 8.0) and analysed on a Perkin Elmer LS50B Luminescence Spectrometer. Solutions were then lyophilized and analysed by agarose gel electrophoresis (1.5%) at constant voltage (126 V) in 1× TBE buffer (pH 8.3) for 1 h. The gel was imaged first, then stained with ethidium bromide solution (1 μg/ml) for 20 min to enable the unlabelled DNA to be visualized.

### Fluorescent labelling of single-stranded DNA probes from exonuclease digestion

AHP-modified or unmodified single-stranded DNA (ssDNA) was prepared by exonuclease digestion of the equivalent double strand (supporting information S9). A total of 1.46 μg of T8 single-stranded DNA or 2.54 μg of T11 single-stranded DNA was mixed with 1× Gotaq green buffer (20 μl) containing 10 nmol of BCN-functionalized fluorophores (either a single fluorophore or a mixture of two different fluorophores, 2 mM stock solution of each dye in DMSO). The labelling reactions were left at 55°C for 1 h and ethanol precipitation of the DNA was used to remove unreacted free dyes. The DNA precipitates were re-dissolved in 1× Gotaq green buffer (10 μl). Half of the solution (5 μl) was mixed with the complementary strand and melting temperature experiments were carried out on a BIO-RAD CFX96 real-time PCR instrument. Labelled ssDNA and re-annealed products were analysed by 1.5 or 2% agarose gel electrophoresis at constant voltage (126 V) in 1× TBE buffer. Template T8 PCR products were also analysed by 12% native polyacrylamide gel electrophoresis (PAGE) at constant voltage (120 V) in 1× TBE buffer (pH 8.3) for 15 h. The gel was initially imaged in a channel appropriate for the dye used (Supplementary Table S4, ESI), then stained with ethidium bromide (1 μg/ml) or SYBR Gold (1×) solution for 20 min to enable the unlabelled DNA to be visualized.

### Dual-labelling of single-stranded DNA probes on streptavidin magnetic beads

PCR was carried out with one unmodified primer and one biotinylated primer using different ratios of AHP dUTP to dTTP (Supporting Information S6). Dynabeads™ M-280 Streptavidin (10 mg/ml, Thermo Fisher Scientific) were washed three times with 2× TE buffer (10 mM Tris–HCl, 1 mM ethylenediaminetetraacetic acid, 2 M NaCl, pH 7.5). AHP–modified amplicons from one PCR reaction (20 μl, T8 products) were mixed with Dynabeads (20 μl, 50 μg) in 2× TE buffer and gently rotated at RT for 30 min. The supernatant was removed and the beads were washed three times with 2× TE buffer. The beads were re-suspended in 20 mM NaOH (20 μl) and rotated at RT for 30 min. The supernatant was removed and the beads were then washed three times with 2× TE buffer and re–suspended in 1× Gotaq green buffer (20 μl) with 5 nmol of a mixture of two BCN-fluorophores (2 mM stock solution of each dye in DMSO). The labelling reactions were heated at 55°C for 1 h, then 70% ethanol was used to wash the beads three times. The beads were re-suspended in formamide (50 μl) and heated at 65°C for 5 min in a heating block. The formamide solutions were analysed by 8% denaturing PAGE or collected and lyophilized. The residues were re-dissolved in 1× Gotaq green buffer (15 μl) and half of the solution (7.5 μl) was mixed with the complementary strand (T8, 1.2 eq) and melting temperature experiments were carried out on a BIORAD CFX96 real-time PCR instrument. The labelled ssDNA and the re-annealed samples were analysed by 12% native PAGE at constant voltage (120 V) in 1× TBE buffer (pH 8.3) for 15 h. Gels were imaged in the channel appropriate for the dye used (Supplementary Table S4, ESI), then stained with 1× SYBR Gold. Fluorescent bands were quantified using ImageJ software.

## RESULTS AND DISCUSSION

### Synthesis of azide-modified triphosphate

**Figure 2.**
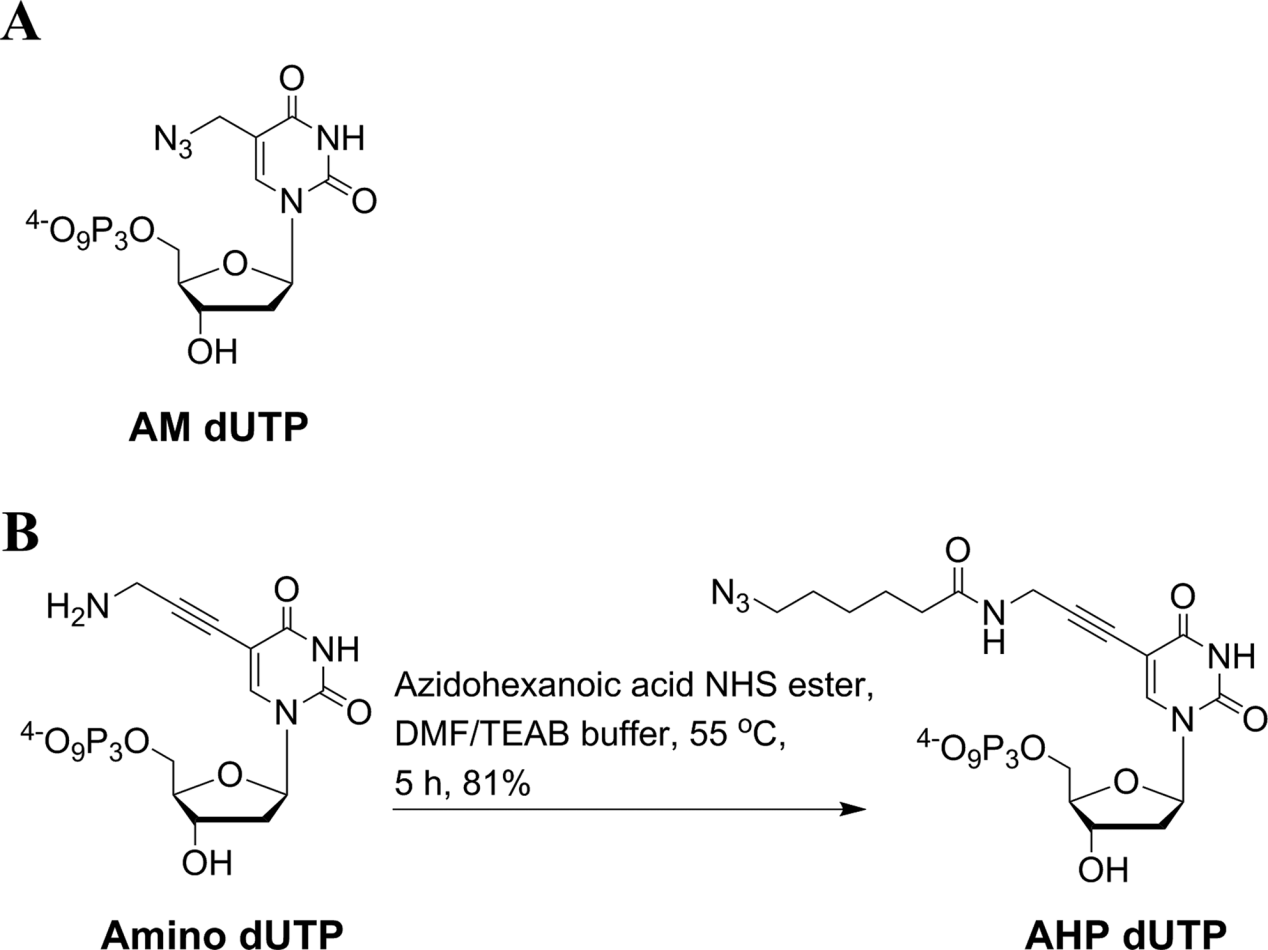
(**A**) Chemical structure of azidomethyl (AM) dUTP. (**B**) Synthesis of azidohexanamidopropargyl deoxyuridine triphosphate (AHP dUTP).

To improve upon the enzymatic incorporation and fluorescent labelling efficiency that was previously achieved by AM dUTP (Figure 2A), (19) and with ease of chemical synthesis in mind, a new azide-modified triphosphate (AHP dUTP) was designed. The AHP dUTP was produced by labelling aminopropargyl dUTP with 6-azidohexanoic acid *N*–hydroxysuccinimide ester (NHS, Figure 2B) (18,19). The aminopropargyl linkage has been shown previously to have good DNA polymerase-compatibility (30–34).

### Primer extension using AHP dUTP

Enzymatic incorporation of AHP dUTP was evaluated using a variety of DNA templates and polymerases. We have used the same DNA templates previously, and they were utilized here to allow direct comparisons to be made (18,19). Template T1 carries three evenly distributed adenines in the extension region and template T2 contains a total of six adenine bases, with four consecutive adenines at the start of the primer extension region (Table 1). The templates are designed to give one-base (T1) or four-base (T2) extension products in the presence of dTTP or AHP dUTP alone (i.e. in the absence of dATP, dCTP and dGTP), whereas full length 11-bases extension products should be obtained in reactions that include a full complement of dNTPs, i.e. dTTP or AHP dUTP in addition to dATP, dCTP and dGTP. To allow visualization of the extension products after analysis by denaturing PAGE, 6-carboxyfluorescein (FAM) was attached to the 5′-end of the primer. Four different polymerases were evaluated in the primer extension reactions—the Klenow large fragment, Gotaq, Therminator™ II and KOD. Klenow large fragment from *Escherichia coli*. is a family-A polymerase that functions efficiently at 37°C. Gotaq (also a family-A polymerase), Therminator™ II and KOD (family-B polymerases) are thermostable enzymes, functioning most efficiently above 60°C. Previous studies on incorporation of modified nucleoside triphosphates have shown that family-B polymerases are more efficient than family-A polymerases (7,30,35).

**Table 1.** Oligonucleotide sequences used in primer extension, reverse transcription and PCR

Code	Sequence (5′-3′)
T1	C**A**GTC**A**CTGT**A**CTGCCGACACACATAACC (DNA template)
T2	C**A**GTC**A**C**AAAA**CTGCCGACACACATAACC (DNA template)
P3	FAM-GGTTATGTGTGTCGGCAG (primer)
T4	C**A**GUC**A**CUGU**A**CUGCCGACACACAUAACC (RNA template)
T5	C**A**GUC**A**C**AAAA**CUGCCGACACACAUAACC (RNA template)
P6	GCATTCGAGCAACGTAAG (PCR primer for T8)
P7	GGTTATGTGTGTCGGCAG (PCR primer for T8)
P6_p_	Phosphate-GCATTCGAGCAACGTAAG (T8 primer for λ-exonuclease digestion)
P7_p_	Phosphate-GGTTATGTGTGTCGGCAG (T8 primer for λ-exonuclease digestion)
P6_b_	Biotin-GCATTCGAGCAACGTAAG (T8 primer for streptavidin magnetic separation)
T8	GGTTATGTGTGTCGGCAGTATTGTCAGTGTGAATTCCAGAGTGTGAGATTGTGTGCTGGCGATCTTACGTTGCTCGAATGC (PCR template)
P9	GTTTGGCTTTAGAGGCTGGAG (PCR primer for T11)
P10	ACTGCAATACGAATAATGGCTAC (PCR primer for T11)
P9_p_	Phosphate-GTTTGGCTTTAGAGGCTGGAG (T11 primer for λ-exonuclease digestion)
P10_p_	Phosphate-ACTGCAATACGAATAATGGCTAC (T11 primer for λ-exonuclease digestion)
T11	plasmid HydGdCTD5 template (sequence in ESI)

FAM is 6-carboxamidohexylfluorescein. The bold **A** shows the incorporation sites of the modified triphosphates.

**Figure 3.**
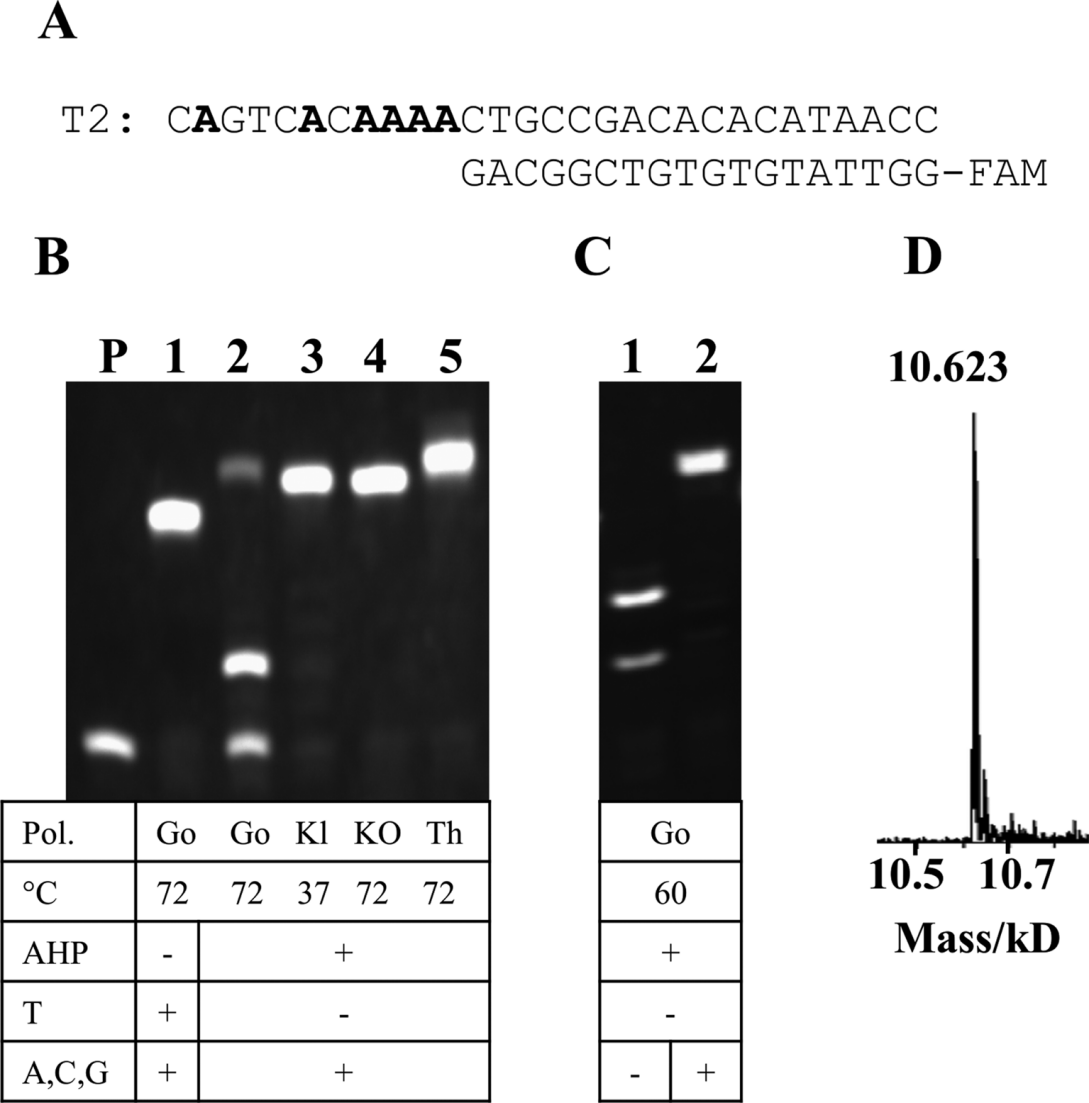
Primer extension using AHP dUTP (1.5 h). (**A**) Template T2 and primer P3. (**B**) Twenty percent denaturing PAGE analysis of reactions using Gotaq (Go, 72°C), Klenow (Kl, 37°C), KOD (KO, 72°C) and Therminator™ II (Th, 72°C) polymerases. Lane P: primer P3. (**C**) Reactions using Gotaq polymerase at 60°C. (**D**) Mass spectrum of AHP-modified fully extended product using Klenow (calculated mass: 10621).

Denaturing PAGE and mass spectrometry (MS) analysis confirmed that all four polymerases successfully incorporate AHP dUTP in the presence of templates T1 and T2 to produce fully extended products (Figure 3, Supplementary Figure S2 and Table S3, ESI). However, in the case of Gotaq with template T2, an intense primer band and a two-base extension product band were observed. Indeed, Gotaq generally gave lower primer extension efficiency with AHP dUTP compared to the other three polymerases at their optimum temperatures (Figure 3B). However, longer incubation times at 72°C enhanced the yields for the Gotaq reactions (Supplementary Figure S3, ESI). Melting temperature (*T*_m_) studies on primer extension products in Gotaq green buffer showed that the partially extended and full length products melt between 62°C and 70°C. Therefore the reaction temperature for Gotaq polymerase was lowered to 60°C. Pleasingly fully extended products were then detected (Figure 3C, lane 2), showing that decreasing the temperature facilitated the reaction, presumably by stabilizing the DNA duplex. For Therminator™ II and Gotaq, one additional adenine nucleobase was incorporated at the end of the extended products (Figure 3B, lanes 2 and 5, confirmed by mass spectrometry). This is a well-known phenomenon.

The kinetic parameters of single AHP dUTP incorporation were studied for Gotaq and KOD polymerases using template T1 at 60°C (Supplementary Figure S4 and Table S2, ESI). For KOD, the relative catalytic efficiency *K*_cat_/*K*_m_ of AHP dUTP to dTTP was 0.91, supporting the observation that AHP dUTP is a suitable substrate for KOD polymerase. For Gotaq polymerase the relative *K*_cat_/*K*_m_ of AHP dUTP to dTTP was 0.08, demonstrating that Gotaq is significantly better at incorporating the unmodified nucleobase. The binding affinity of AHP dUTP for Gotaq polymerase (*K*_m_ = 164 ± 20 μM) was 2.7× higher than for dTTP (*K*_m_ = 61 ± 10 μM), but the turnover rate of AHP dUTP (*K*_cat_ = 0.52 ± 0.02 s^−1^) was 4.6× slower than for dTTP (*K*_cat_ = 2.40 ± 0.16 s^−1^); this is consistent with the lower incorporation efficiency of AHP dUTP that was observed with Gotaq polymerase.

### Synthesis of BCN fluorophores and fluorescent labelling of primer extension products

**Figure 4.**
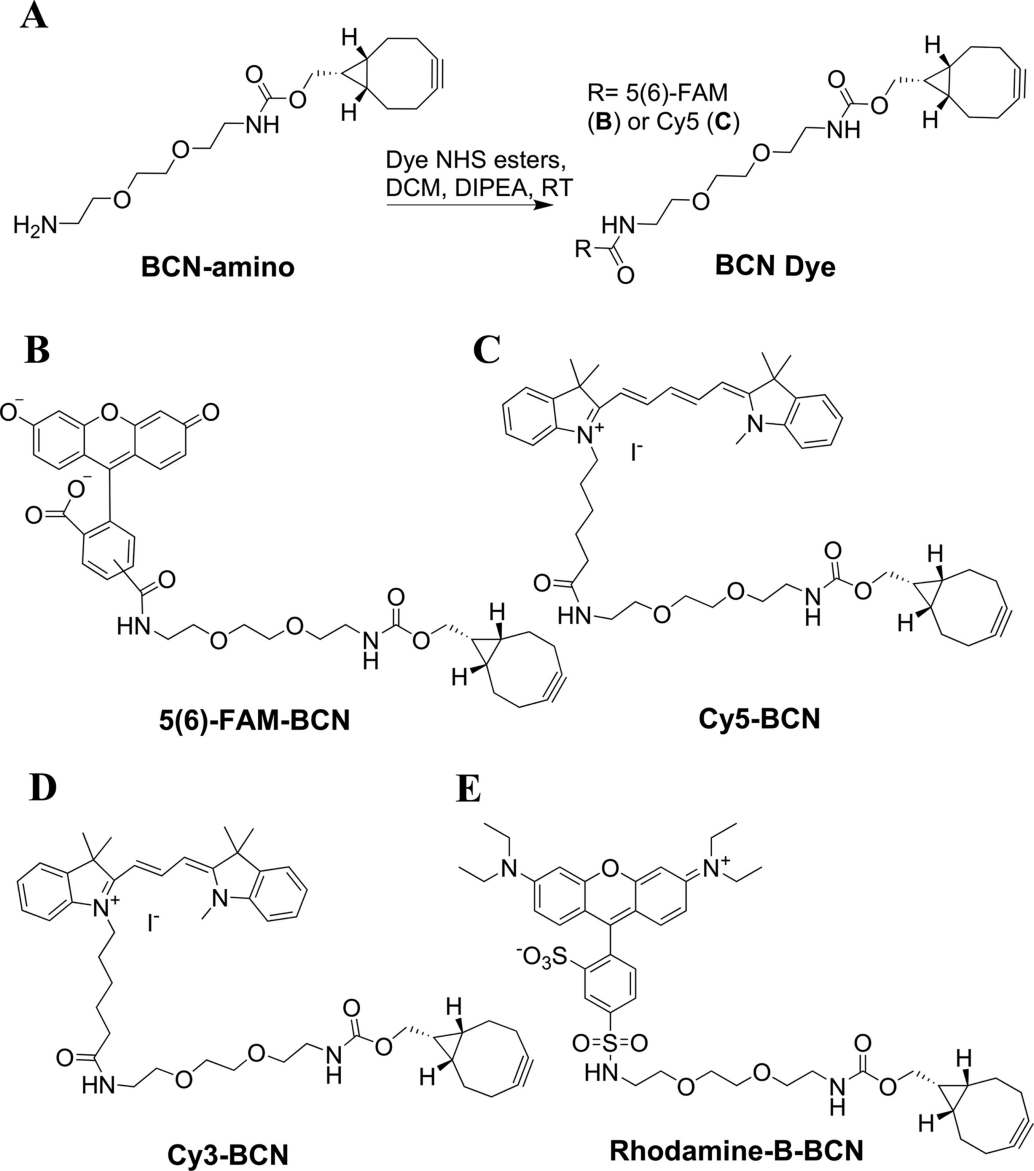
(**A**) Synthesis of BCN-functionalized fluorophores. (**B**) 5(6)-Carboxyfluorescein (FAM)-BCN (for ease of description 5(6)-FAM-BCN is written as FAM-BCN subsequently). (**C**) Cy5-BCN. (**D**) Cy3-BCN (19). (**E**) Rhodamine-B-BCN.

**Figure 5.**
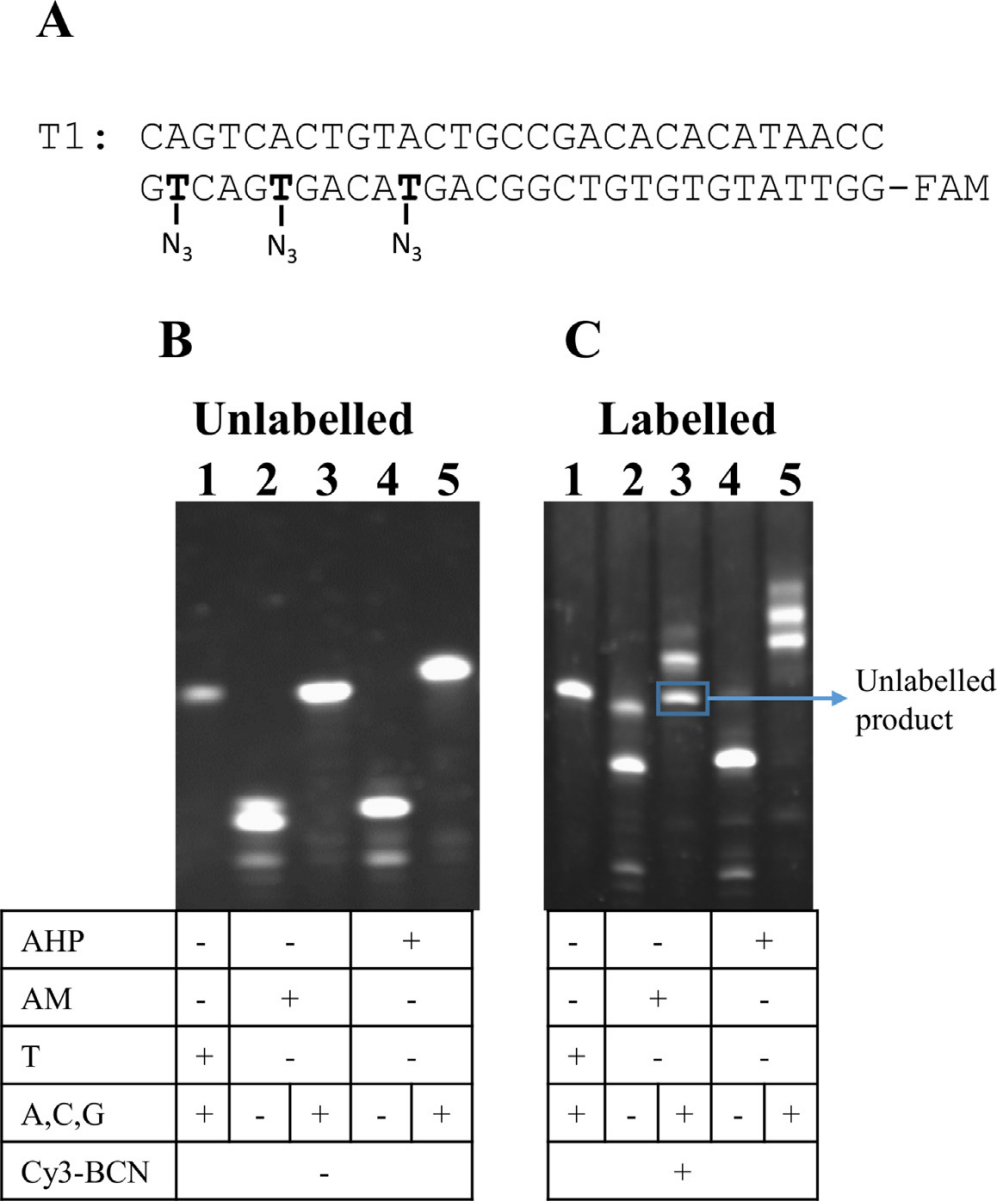
Fluorescent labelling of AM and AHP-modified primer extension products. (**A**) Fully extended products from primer P3 and template T1. (**B** and **C**) Twenty percent denaturing PAGE analysis of samples before labelling and after labelled with Cy3-BCN at RT for 2 h. The unmodified product was used as a negative control.

After incorporation of AHP dU, BCN functionalized fluorophores were introduced into the azide-modified DNA strands via the SPAAC reaction. BCN-functionalized 5(6)-FAM and Cy5 dyes were synthesized by coupling BCN amino-derivative with NHS esters of 5(6)-FAM and Cy5 (Figure 4A–C). Cy3-BCN (Figure 4D) was initially used to label the long chain azide products obtained from AHP dUTP incorporation and also to label the short chain azide products from AM dUTP (19). The latter was carried out to allow comparison with our previous work (19). The fully extended products from template T1 containing AHP dU exhibited much greater labelling efficiency than the AM dU product as judged by the presence of remaining unlabelled products in the latter (Figure 5C, lanes 3 and 5). AHP has a much longer linker between the azide and the uracil base, which protrudes out from the major groove of the DNA duplex; it will therefore experience less steric hindrance during the SPAAC labelling reaction.

The labelling efficiency of all three fluorophores (Cy3-BCN, FAM-BCN and Cy5-BCN) was then studied using the AHP-modified full length extension product from template T1 (Supplementary Figure S5, ESI). Denaturing PAGE analysis indicated that the labelling of AHP-DNA with Cy3-BCN was more efficient than with Cy5-BCN or FAM-BCN. The negative charge of the FAM moiety most likely decreases the reactivity of BCN toward the azides that are located close to the anionic phosphodiesters in the DNA major groove. However, the FAM-labelled product exhibited better aqueous solubility. The FAM-labelled product was analysed by mass spectrometry and the expected mass peaks were observed for incorporation of 2 × FAM-BCN (calc. 11 455, found 11 457) and 3 × FAM-BCN (calc. 12 137, found 12 139). Much weaker bands for the products with three Cy5 dyes were observed (Supplementary Figure S5, ESI), due to either fluorescence quenching or solubility problems caused by the hydrophobicity of Cy5.

### Reverse transcription using AHP dUTP

**Figure 6.**
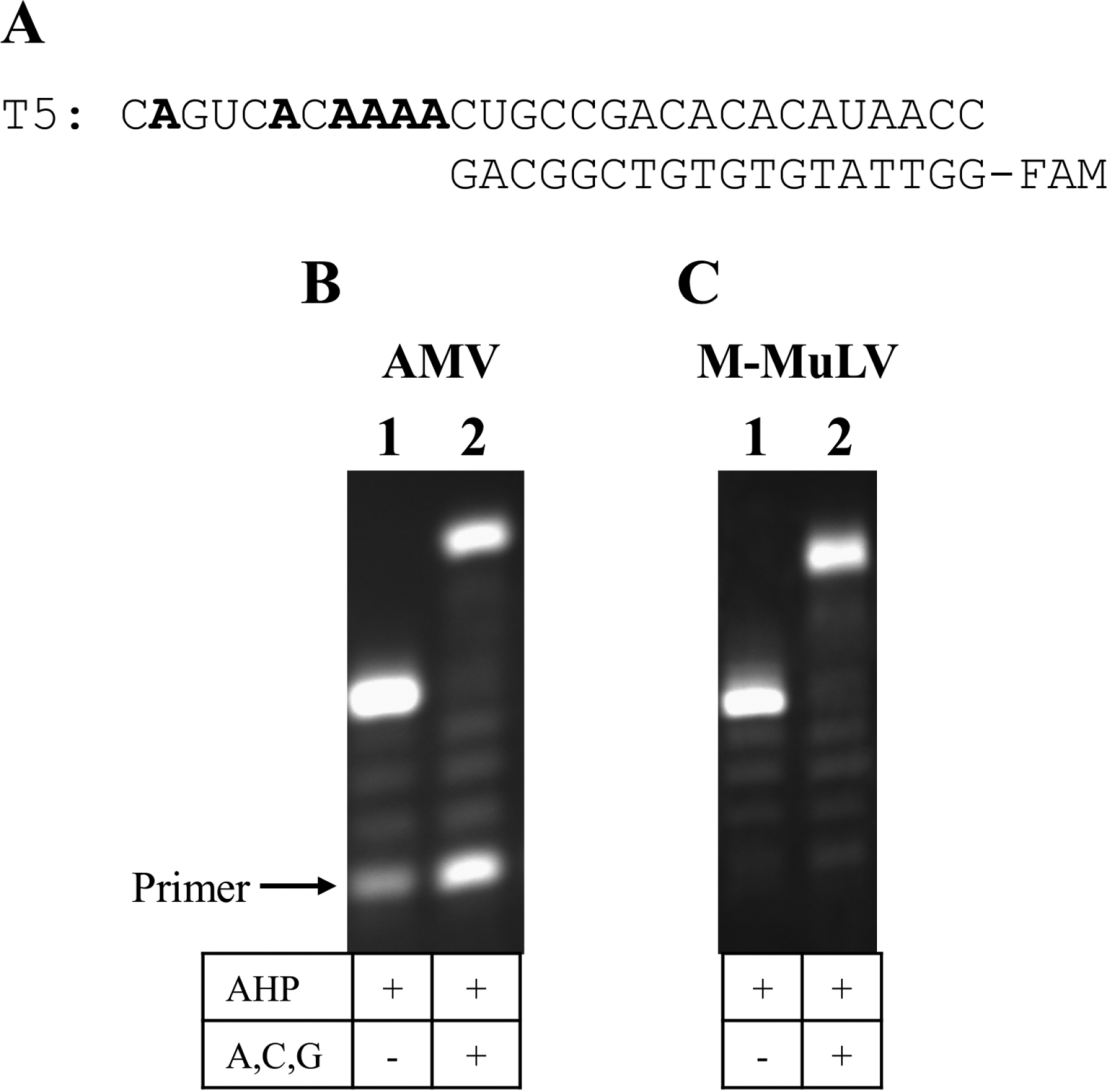
Reverse transcription using AHP dUTP. (**A**) RNA template T5 with primer P3. (**B** and **C**) Twenty percent denaturing PAGE analysis of reactions using AMV and M-MuLV (RNase H-) reverse transcriptases at 42°C for 15 h.

Reverse transcription was carried out to investigate the compatibility of AHP dUTP with RNA-dependent polymerases, and to evaluate the synthesis of modified DNA from RNA templates. Two synthetic RNA templates were used, carrying the equivalent sequence information to the above DNA templates (Table 1, T4 and T5); and two reverse transcriptase enzymes, from Moloney Murine Leukemia Virus (M-MuLV, RNase H^−^) and Avian Myeloblastosis Virus (AMV), were tested. AHP dUTP was incorporated successfully in all cases, affording fully extended products (Figure 6 and Supplementary Figure S6, ESI). The M-MuLV (RNase H^−^) reactions gave near-quantitative yields of full length products, whereas unextended primer bands were observed when AMV was used. This indicates that M-MuLV (RNase H^−^) is the preferred choice for RNA-dependent DNA synthesis using AHP dUTP.

### PCR amplification using AHP dUTP

**Figure 7.**
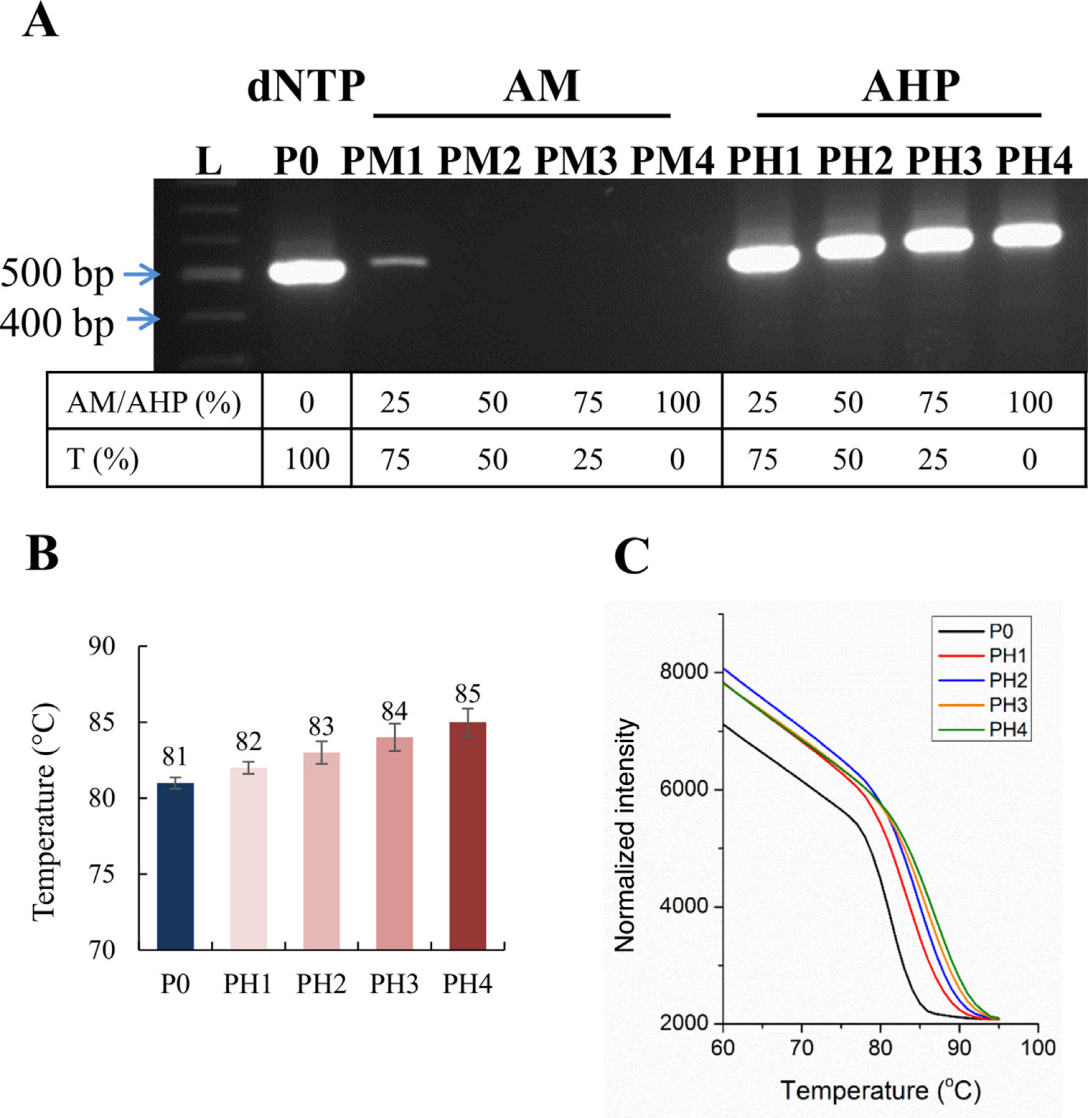
PCR amplification using template T11, primers P9, P10 and KOD polymerase with different ratios of azide dUTPs to dTTP (10 nmol in total of azide dUTP/dTTP + 10 nmol each of dATP, dCTP and dGTP). (**A**) 1.5% agarose gel analysis. AM lanes: AM dUTP reactions; AHP lanes: AHP dUTP reactions. Lane L: 100 bp ladder. (**B**) Bar chart showing melting temperatures of AHP-modified amplicons. (**C**) Fluorescence melting curves of AHP-modified amplicons.

The above primer extension and reverse transcription studies demonstrate that AHP dUTP is accepted by a variety of polymerases to provide an efficient method to introduce clickable azide modifications into DNA. For applications which have limited quantities of available template, or require densely modified or longer labelled products, PCR is a viable option to introduce the azide modification into both strands of the DNA duplex. The compatibility of AHP dUTP with PCR was initially explored using the 81-mer DNA template T8 and Gotaq polymerase. Encouragingly, using 100% of modified AHP dUTP (i.e. no dTTP) in combination with dATP, dCTP and dGTP, the desired PCR amplicon was produced (Supplementary Figure S7, ESI). A longer 523-bp template (T11) from the hydGΔCTD plasmid containing 335 A-T base pairs was then amplified using KOD polymerase, which is more efficient than Gotaq. Different ratios of AHP dUTP mixed with natural dTTP were used to control the number of reactive groups introduced into the PCR products. Real-time PCR reactions were carried out and subsequently analysed by fluorescent melting experiments. Pleasingly the PCR reaction with 100% AHP dUTP gave an intense product band (Figure 7A, lane PH4). Azide-modified products were obtained more efficiently with AHP dUTP compared to AM dUTP (Figure 7A), possibly due to the duplex destabilizing effect of AM dUTP (19). Consistent with this hypothesis is the observation that amplification efficiency in the presence of AM dUTP was improved by doubling the MgCl_2_ concentration to increase duplex stability (19). The melting temperatures of the AHP-modified PCR products increased progressively with increasing ratios of AHP dUTP to dTTP (Figure 7B) due to the influence of the alkyne linkages which stabilize DNA duplexes by enhanced π-stacking.

To evaluate the fidelity of PCR using AHP dUTP, the PCR products (523 bp) from template T11 (with varying ratios of AHP dU to dT) were sequenced directly by the automated Sanger method. Remarkably the 25% AHP-modified PCR product (PH1) was fully read through during the sequencing reaction. However, the sequencing reactions terminated earlier when the number of azide modifications in the PCR products was increased. To circumvent this problem the AHP-functionalized products were used as templates in a second-round of PCR using all four natural triphosphates. All these unmodified PCR products gave 100% correct sequencing when compared to the original plasmid template (ESI). This demonstrates that AHP dU is an excellent analogue of thymidine in terms of its Watson–Crick base pairing and encoding properties.

### Fluorescent labelling of double-stranded DNA probes

**Figure 8.**
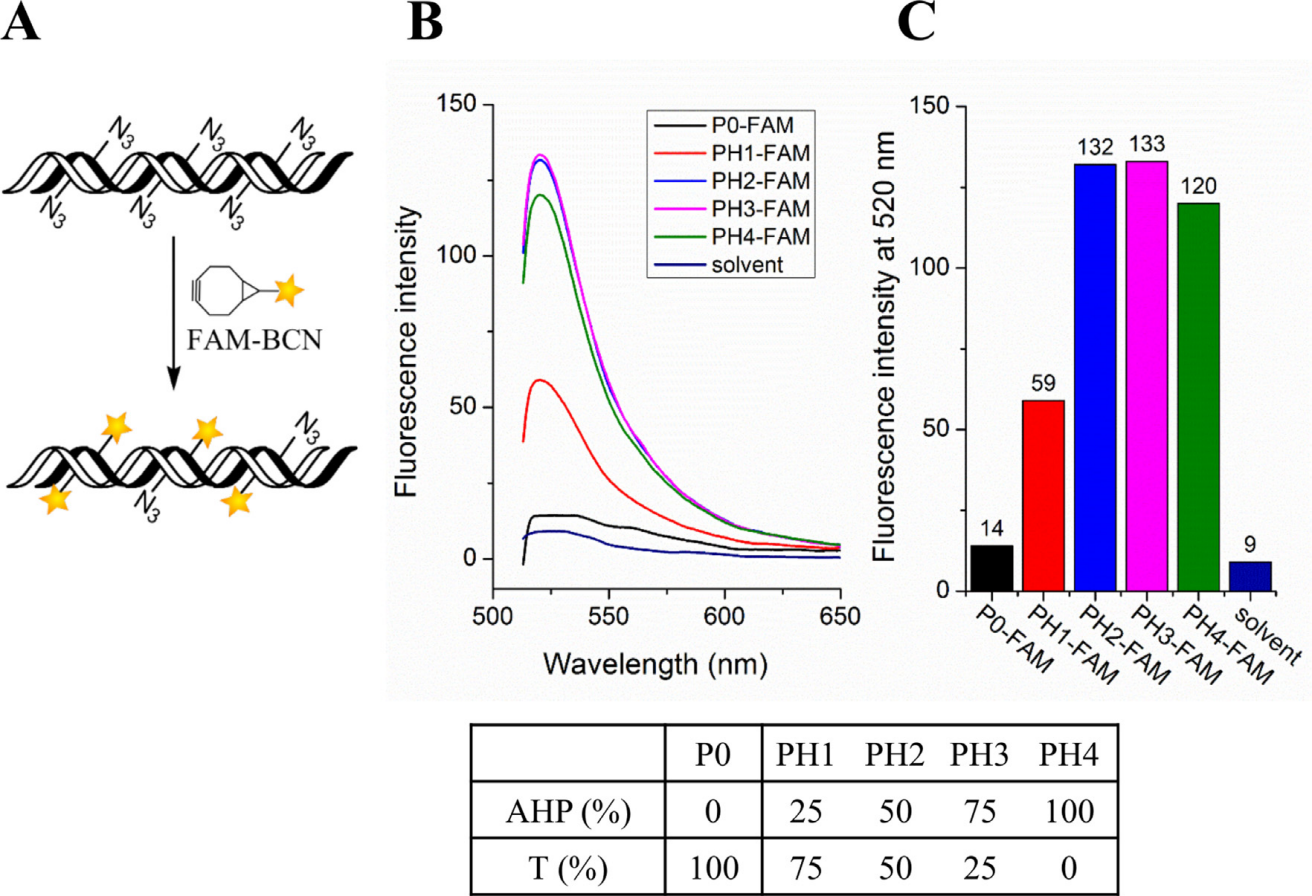
Fluorescent labelling of PCR amplicons from template T11 containing different percentages of AHP dU. (**A**) Scheme for FAM-BCN labelling. (**B**) Fluorescence emission spectra of labelled PCR amplicons in TEAB buffer, excited at 480 nm. (**C**) Fluorescence intensity (relative fluorescence units, RFU) at 520 nm for each sample from B. Solvent: 75 mM TEAB buffer (pH 8.0).

The azide-functionalized T11-template double-stranded PCR products were labelled with Cy3-BCN or FAM-BCN in 30 min at RT (Figure 8 and Supplementary Figure S9, ESI). The labelled products showed intensely fluorescent bands without additional staining, and displayed lower electrophoretic mobility than the unlabelled PCR products due to the bulky nature of the aromatic dyes (Supplementary Figure S9, ESI). The 50 or 75% AHP dU-modified PCR product was labelled efficiently and gave the strongest fluorescent signals. When all dT sites were replaced with AHP dU, the fluorescent signal decreased, presumably due to collisional quenching between the fluorophores (Figure 8). This was more striking for Cy3 and Cy5 labelling (Supplementary Figure S9 and S10, ESI), and is consistent with reports on fluorescent labelling of amino functionalized RNA strands (10). These observations emphasize the importance of carefully evaluating fluorescence as a function of labelling density when synthesizing DNA probes. The highly efficient SPAAC labelling strategy described here has the potential to be used widely to prepare labelled fluorescent (or other modified) probes of controlled label density.

### Dual-colour labelling of double-stranded DNA probes

**Figure 9.**
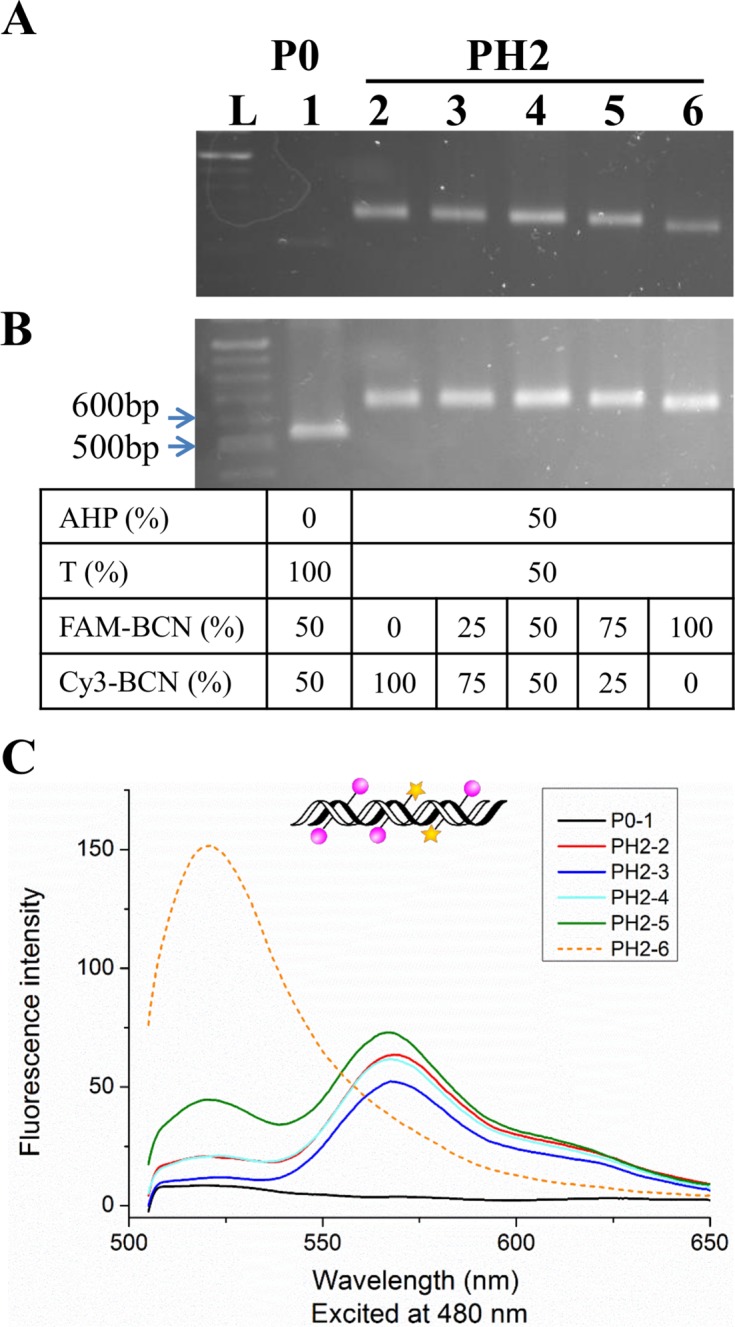
Dual labelling of 50% AHP-modified PCR amplicons from template T11 with different combinations of Cy3-BCN (100–0%) and FAM-BCN (0–100%). (**A** and **B**) 1.5% agarose gel analysis visualized on a transilluminator before staining and after staining with ethidium bromide. (**C**) Fluorescence spectra of labelled PCR amplicons in TEAB buffer (pH 8.0, samples were also analysed by agarose gel, shown in A and B), excited at 480 nm.

Fluorophore-labelled oligonucleotides are commonly used as sequence-specific probes for fluorescence *in situ* hybridization detection of DNA and RNA. However, in order to detect multiple targets simultaneously, several different coloured fluorescent probes are required, and the number of fluorescent dyes that can be used for nucleic acid detection is limited by spectral overlap. In principle one way to overcome this problem is to introduce multiple fluorophores into individual probes to generate a cocktail of probes with different (unique) fluorescence spectra (7). This could be achieved by attaching orthogonal reactive groups to different dNTPs and incorporating them by PCR, followed by fluorescent labelling. However, this would require the synthesis of different modified nucleotides (36). A simpler strategy, described here, is to use a DNA probe labelled with a single modified triphosphate (AHP dUTP) and to label the probe with a mixture containing two different fluorophores. To achieve high labelling efficiency and minimize fluorescent quenching, the 50% AHP-modified PCR products (PH2) were used in dual-fluorophore labelling with varying ratios of Cy3-BCN and FAM-BCN. The electrophoretic mobility of products containing different ratios of Cy3 and FAM was found to vary due to the different charge states of the fluorophores (Figure 9A and B). To detect the fluorescent signals of both dyes, the samples were excited at 480 nm. When the ratio of FAM-BCN to Cy3-BCN in the labelling reaction was varied from 100 to 75%, the fluorescence spectra exhibited different characteristics, with the ratio of the FAM and Cy3 emissions changing significantly (Figure 9C). This technique enables the production of diverse fluorescent labelled products from a single DNA template simply by mixing the two dyes in different ratios. It could be extended to a 3-dye multiple probe system by varying the ratios of the three dyes, and has potential for the detection of multiple gene sequences simultaneously.

### Synthesis of fluorescent single-stranded DNA probes via exonuclease digestion

**Figure 10.**
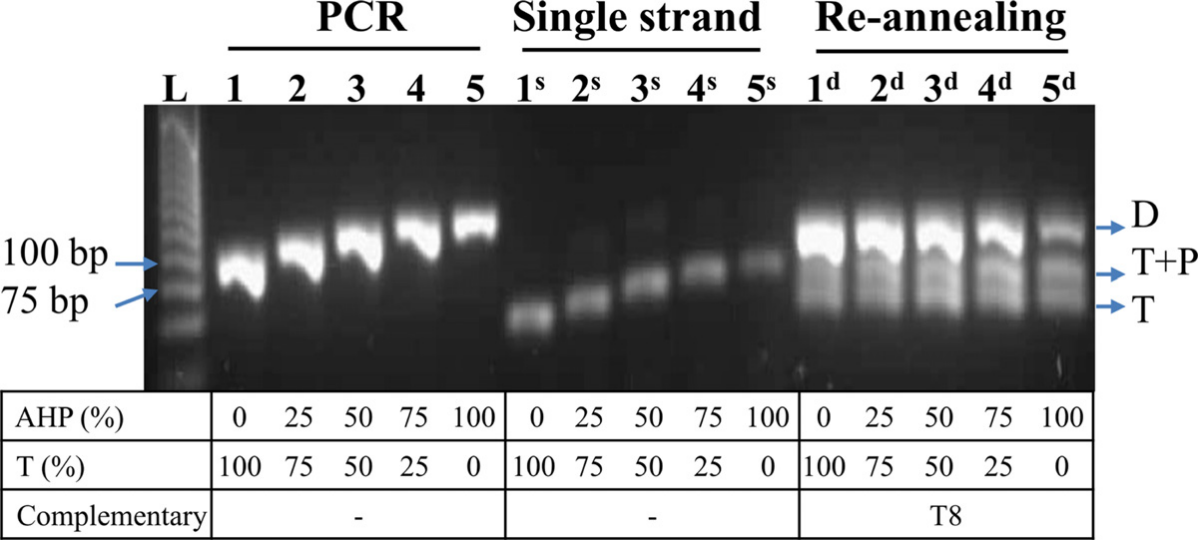
λ-exonuclease digestion of PCR amplicons (81 bp, primer P6 and P7p) containing different percentages of AHP dU modifications and re-annealing of the modified single strands to complementary template T8. 2% agarose gel analysis containing SYBR Gold stain. PCR lanes: PCR amplicons; single strand lanes: PCR amplicons after exonuclease digestion; re-annealing lanes, ssDNA re-annealed with T8. Lane L: 25 bp ladder. D = duplex products; T + P = excess primer annealed to template T8; T = template T8.

In some (but not all) DNA or RNA hybridization-detection applications fluorescent probes are required in a single-stranded form. To this end we investigated the conversion of the above azide-functionalized PCR amplicons to single strands. Two methods were compared: (i) λ-exonuclease digestion of one strand of a conventional PCR product; and (ii) λ-exonuclease digestion of one DNA strand after asymmetric PCR (37–40). In both cases one of the PCR primers was synthesized with a 5′-phosphate group which makes its extension product a better target for λ-exonuclease digestion than the 5′-unphosphorylated complementary strand. Various AHP-modified DNA single strands were successfully obtained by these methods (Figure 10 and Supplementary Figure S11, ESI). As expected, the PCR products with a higher percentage of azide modifications were slightly more resistant to exonuclease digestion compared to the unmodified products, and the 100% AHP dU PCR products (523 bp) had to be treated with λ-exonuclease multiple times to obtain single-stranded products. However, it was gratifying to find that in most cases the digestion proceeded smoothly. This is presumably because the azide modification is sterically undemanding and therefore does not have a major inhibitory effect on λ-exonuclease.

**Figure 11.**
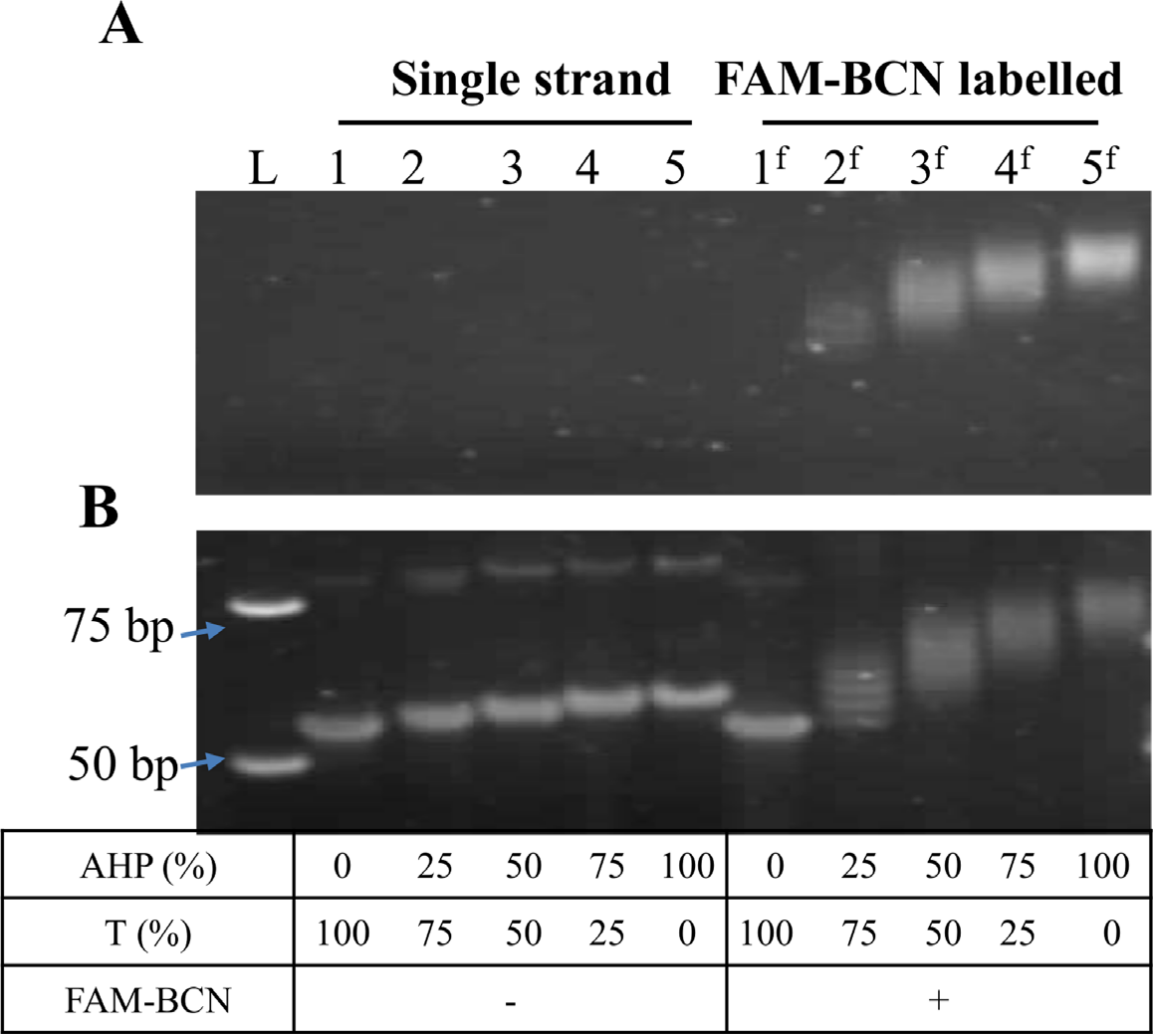
Fluorescent labelling of ssDNA (81-mer) containing different percentages of AHP dU. (**A** and **B**) 12% native PAGE analysis visualized on a transilluminator, before staining and after staining with SYBR Gold. Single strand lanes: single-stranded products from exonuclease digestion; FAM-BCN labelled lanes: ssDNA labelled with FAM-BCN at 55°C for 1 h. Lane L: 25 bp ladder.

The AHP-modified single-stranded DNAs were labelled with FAM-BCN at 55°C. Fluorescently labelled products were clearly observed without staining the gel (Figure 11, FAM-BCN labelled lanes) and were successfully re-annealed with their complementary strand to afford fluorescent double-stranded products (Supplementary Figure S12, ESI). The fluorescence intensity of the re–annealed duplexes increased with higher numbers of AHP dU modifications, confirming that DNA probes with large numbers of bulky fluorophores can form stable duplexes, and are therefore suitable for fluorescence hybridization applications. The dual labelling strategy was also evaluated for the AHP-modified ssDNA (81-mer) using Rhodamine-B-BCN and FAM-BCN (Figure 4B and E). Single-stranded probes with different fluorescent profiles were successfully obtained (Supplementary Figure S14, ESI).

### Synthesis of dual labelled single-stranded DNA probes via streptavidin magnetic bead separation

The previously described fluorescent labelling of ssDNA generated by exonuclease digestion of APCR or PCR products involves multiple purification procedures. To simplify the digestion and labelling strategy, streptavidin-coated magnetic bead separation was also evaluated (41,42). AHP-modified PCR amplicons were obtained using template T8, 5′-biotinylated primer P6_b_ and unmodified primer P7. After binding and denaturation, the un-biotinylated strand was liberated into solution, while the biotinylated complementary single strand remained attached to the magnetic beads. The biotinylated strand was selected for the dual-labelling reaction as it can be purified simply by washing the streptavidin magnetic beads to remove excess reagents. Initial dual-labelling of the 50 or 100% AHP-modified products using different combinations of FAM-BCN and Cy3-BCN (or Cy5-BCN) afforded fluorescent single stranded probes, which were annealed with the unmodified complementary strand T8 to form a hybrid duplex with distinctive fluorescence properties (Supplementary Figure S15 and S16, ESI). Interestingly the FAM signal dropped sharply when mixtures of FAM-BCN and Cy dye-BCN derivatives were used. This is consistent with our observations on dual fluorophore-labelled AHP-modified PCR amplicons in which both strands are labelled (Figure 9). This may be because the BCN derivative of FAM, which is negatively charged, reacts slowly with azido-DNA due to unfavourable electrostatic interactions between FAM and DNA. Greater reactivity would be expected for positively charged Cy-dyes which will be attracted to DNA. Since a similar rapid drop in FAM fluorescence is observed for mixtures of FAM/Cy3 (Supplementary Figure S15) and FAM/Cy5 (Supplementary Figure S16) the weak FAM signal cannot be explained entirely by FRET.

**Figure 12.**
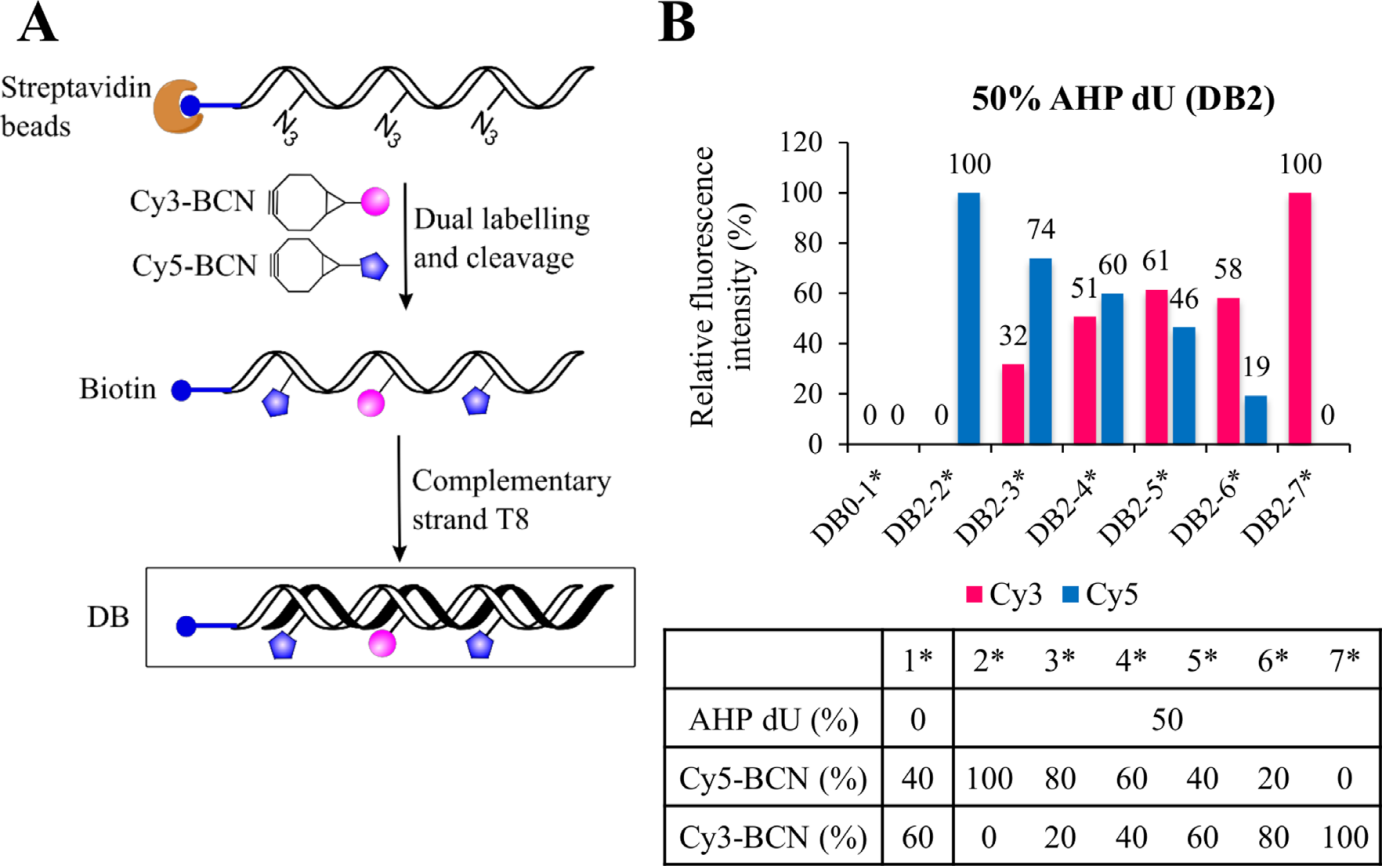
Dual labelling of 50% AHP-modified single strands from streptavidin magnetic bead separation with different combinations of Cy5-BCN (100% to 0%) and Cy3-BCN (0% to 100%). (**A**) Scheme for dual labelling and re-annealing. (**B**) Relative fluorescence intensity for the re-annealed duplex bands (DB2) quantified by gel analysis in Cy5 and Cy3 channels (for native PAGE analysis see Supplementary Figure S17, ESI).

Next, dual-labelling of DNA using Cy3-BCN and Cy5-BCN was carried out. No fluorescent bands were observed for the dual labelled single strands, presumably due to the collisional fluorescence quenching between each fluorophore in the unstructured ssDNA (Supplementary Figures S17 and S18, ESI). In contrast, when annealed to the complementary T8 strand, the labelled fluorophores are held apart by the rigid duplex, reducing collisional quenching and giving rise to intense fluorescent bands. This favourable ‘off-on’ system that responds to duplex formation should be highly convenient for hybridization-based applications as it will reduce the background signal from the unhybridized probe. Importantly, fluorescence measurements on Cy3/Cy5 labelled duplexes revealed a progressive decrease in Cy5 signal when the percentage of Cy5-BCN was varied between 100 and 0%, while the Cy3 fluorescence intensity increased in proportion to the percentage of Cy3-BCN used, and FRET from Cy3 to Cy5 did not seem to strongly suppress the Cy3 signal (Figure 12). This clearly demonstrates the feasibility of making fluorescent probes with subtly different fluorescence characteristics (unique spectra) using only two different fluorophore labels.

## CONCLUSIONS

Primer extension, reverse transcription and PCR proceeded with remarkable efficiency using AHP dUTP-modified nucleotides. The long alkyl chain and ethynyl attachment to the nucleobase in AHP dUTP stabilizes double stranded DNA, accounting in part for its favourable properties as a substrate for polymerase enzymes. In addition to its quantitative enzymatic incorporation, AHP dUTP exhibits high reactivity (and therefore excellent labelling efficiency) with BCN fluorescent dyes, and is clearly superior to the short chain AM dUTP. Using AHP dUTP we have devized an efficient method to prepare dual-colour fluorescent probes (single or double-stranded) with subtly different emission spectra for detection of target DNA or RNA. Our fluorescent dye incorporation studies emphasize the importance of carefully evaluating fluorescence signal as a function of labelling efficiency; high labelling density reduces fluorescent output by quenching mechanisms. The methodology described in this paper could be applied in many areas of biology and nanotechnology including fluorescence *in situ* hybridization to image DNA and RNA. It will also have applications in the synthesis of aptamer libraries via SELEX (43,44) or by the recently developed click SELEX methodology (45). Work is also ongoing in our laboratory to investigate the use of AHP-dU in cellular imaging of genomic DNA, and single molecule experiments are also being pursued (46).

## Supplementary Material

SUPPLEMENTARY DATA
