# Supplementary material for: Efficient enzymatic synthesis and dual-colour fluorescent labelling of DNA probes using long chain azido-dUTP and BCN dyes
Source: Nucleic Acids Res. 2016 Jan 26;44(8):e79. doi: 10.1093/nar/gkw028 (PMC4856977; doi:10.1093/nar/gkw028)
Supplement: SUPPLEMENTARY DATA [file supp_44_8_e79__index.html]

Efficient enzymatic synthesis and dual-colour fluorescent labelling of DNA probes using long chain azido-dUTP and BCN dyes — Efficient enzymatic synthesis and dual-colour fluorescent labelling of DNA probes using long chain azido-dUTP and BCN dyes — SUPPLEMENTARY DATA 

# Efficient enzymatic synthesis and dual-colour fluorescent labelling of DNA probes using long chain azido-dUTP and BCN dyes

## SUPPLEMENTARY DATA

- SUPPLEMENTARY DATA
