# Supplementary material for: Efficient enzymatic synthesis and dual-colour fluorescent labelling of DNA probes using long chain azido-dUTP and BCN dyes
Source: Nucleic Acids Res. 2016 Jan 26;44(8):e79. doi: 10.1093/nar/gkw028 (PMC4856977; doi:10.1093/nar/gkw028)
Supplement: SUPPLEMENTARY DATA [file supp_gkw028_supporting-information-final.docx]

Supporting Information

**Efficient Enzymatic Synthesis and Dual-Colour Fluorescent Labelling of DNA Probes Using Long Chain Azido-dUTP and BCN Dyes**

Xiaomei Ren^1^, Afaf H. El-Sagheer^1, 2^ and Tom Brown^1^*

^1^ Department of Chemistry, University of Oxford, Chemistry Research Laboratory, 12 Mansfield Road, Oxford, OX1 3TA, UK

^2^ Chemistry Branch, Department of Science and Mathematics, Faculty of Petroleum and Mining Engineering, Suez University, Suez, 43721, Egypt

* To whom correspondence should be addressed. Tel: +44 1865 275413; Fax: +44 1865 275 410; E-mail: [tom.brown@chem.ox.ac.uk](mailto:tom.brown@chem.ox.ac.uk)

Contents

[S1: Chemical synthesis: general information 3](#_Toc438554486)

[S2: General method for oligonucleotide synthesis and purification 7](#_Toc438554487)

[S3: Primer extension reactions 9](#_Toc438554488)

[S4: Reverse transcription 12](#_Toc438554489)

[S5: Mass spectrometry analysis of primer extension and reverse transcription products 14](#_Toc438554490)

[S6: PCR amplification 14](#_Toc438554491)

[S7: Sequencing of PCR products (T11) 15](#_Toc438554492)

[S8: Synthesis of fluorescent double-stranded probes (T11) 18](#_Toc438554493)

[S9: Synthesis of fluorescent single-stranded probes 19](#_Toc438554494)

[References: 27](#_Toc438554495)

# S1: Chemical synthesis: general information

All reagents were purchased from Sigma-Aldrich, Alfa Aesar, Acros Organics, SynAffix or Fisher Scientific and used without purification with the exception of dichloromethane (DCM) and *N*, *N*‑diisopropylethylamine (DIPEA) which were dried over activated molecule sieves overnight. Thin layer chromatography (TLC) was performed using Merck Kieselgel 60 F24 silica gel plates (0.22 mm thickness, aluminium backed) and compounds were visualised by irradiation at 254 nm or by staining with anisaldehyde. Column chromatography was carried out under slight pressure using Merck Kieselgel Si 60 (40-63 micron) silica.

^1^H NMR spectra were measured at 500 MHz on a Bruker AVIII500 spectrometer, or on a Bruker DRX500 spectrometer. The ^13^C NMR spectra were measured at 126 MHz on a Bruker DRX500 spectrometer, or AVII500 spectrometer. The ^31^P NMR spectra were recorded at 202 MHz on a Bruker DRX500 spectrometer. Chemical shifts are given in ppm, and *J* values are quoted in Hz. ^1^H NMR spectra were internally referenced to the appropriate undeuterated solvent residual signal, assignment of the compounds was aided by COSY (^1^H-^1^H), HSQC-DEPT and HMBC (^1^H-^13^C) experiments.

All low-resolution mass spectra were recorded using electrospray ionisation on a Micromass platform 1 spectrometer in HPLC grade acetonitrile or methanol. The nucleoside triphosphate and oligonucleotides were characterised by negative-mode electrospray HPLC-mass spectrometry (HPLC‑MS), using an Acquity UPLC system with a BEH C18 1.7 μm column (Waters) and a Bruker micro-TOF mass spectrometer. A gradient of methanol in triethylamine (TEA) and hexafluoroisopropanol (HFIP) was employed, increasing from 0% to 70% buffer B over 8 min, with a flow rate of 0.2 mL/min (buffer A: 8.6 mM TEA, 200 mM HFIP in 5% methanol/water; buffer B: 20% buffer A in Methanol). Raw data were processed and deconvoluted using the Data Analysis function of the Bruker Daltronics Compass^TM^ 1.3 software package.

#### 5-(3-(6-azidohexanamido)prop-1-yn-1-yl)-2ʹ-deoxyuridine-5ʹ-*O*-triphosphate (AHP dUTP)

The freeze-dried amino dUTP ([1](#_ENREF_1),[2](#_ENREF_2)) (111 OD_290_, 9.25 μmol, 1.0 eq) was dissolved in 1 M triethylammonium bicarbonate buffer (TEAB, 300 µL, pH 7.5). The 6-azidohexonic acid NHS ester ([3](#_ENREF_3)) (6 mg, 24 μmol, 2.6 eq) in DMF (300 µL) was added to the triphosphate solution. The mixture was kept at 55 ºC for 4 h and the solvent was removed *in vacuo.* The product was purified by RP-HPLC (Gemini-NX 10u C18 110A AXIA, 250 × 21.2 mm; eluent A: 0.1 M TEAB buffer (pH 7.5), B: 50% acetonitrile in 0.1 M TEAB buffer (pH 7.5), 15% to 60% buffer B in 50 min and monitored by UV absorption at 305 nm). Pure AHP dUTP (90 OD_290_, 7.5 μmol) was obtained in 81% yield. M_w_ = 660.36 g/mol (Chemical Formula: C_18_H_27_N_6_O_15_P_3_).


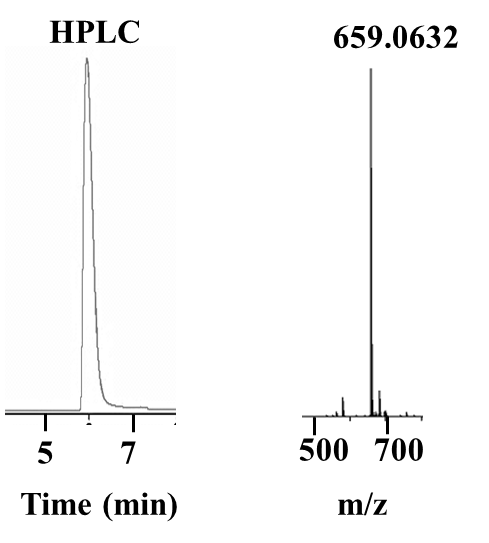


Figure S1. The HPLC trace and mass spectrum of AHP dUTP (calc. 659.0669 [M-H^+^], found 659.0632).

^1^H NMR (500 MHz, D_2_O) δ 8.08 (s, 1 H, H-6), 6.24 (t, *J* = 6.6 Hz, 1 H, H-1ʹ), 4.66 – 4.57 (m, 1 H, H-3ʹ), 4.28 – 4.10 (m, 5 H, H-4ʹ, H-5ʹ, H-9 ), 3.26 (t, *J* = 6.9 Hz, 2 H, H-16), 3.15 (q, *J* = 7.3 Hz, Et_2_N^+^C***H***_2_CH_3_), 2.39 – 2.29 (m, 2 H, H-2ʹ), 2.26 (t, *J* = 7.3 Hz, 2 H, H-12), 1.65 – 1.49 (m, 4 H, H-13, H-15), 1.34 (tt, *J* = 9.1, 6.3 Hz, 2 H, H-14), 1.23 (t, *J* = 7.3 Hz, Et_2_N^+^CH_2_C***H***_3_)

^13^C NMR (126 MHz, D_2_O) δ 177.2 (C=O), 144.9 (C6), 99.5 (C≡C), 89.7 (C≡C), 85.9 and 85.8 (C1′, C4′), 75.0 (C≡C), 70.3 (C3′), 65.4 (C5′), 51.4 (C16), 47.0 (Et_3_N^+^***C***H_2_CH_3_), 39.1 (C2′), 35.8 (C12), 30.0 (C9), 28.1 (C15), 26.3 (C14), 25.2 (C13), 8.6 (Et_2_N^+^CH_2_***C***H_3_)

^31^P NMR (202 MHz, D_2_O) δ -5.2 (d, *J* = 20 Hz, P_γ_), -10.1 (d, *J* = 20 Hz, P_α_), -21.3 (app t, *J* = 20 Hz, P_β_)

#### Cy5-BCN

Cy5 NHS ester ([4](#_ENREF_4)) (30 mg, 0.049 mmol, 1.0 eq) was dissolved in anhydrous DCM (2.0 mL), anhydrous DIPEA (30 μL, 0.17 mmol, 3.5 eq) and BCN amine (25 mg, 0.077 mmol, 1.6 eq, purchased from SynAffix) were added. The reaction mixture was stirred at room temperature for 7 h, diluted with DCM (7 mL) and washed with saturated aqueous potassium iodide (10 mL). The organic layer was dried over sodium sulphate, filtered and evaporated. The crude solid was purified by column chromatography on silica (0% to 10% MeOH in DCM). Cy5-BCN (27 mg, 0.030 mmol) was obtained in 60% yield as a dark blue salt. M_w_ = 916.40 g/mol (Chemical Formula: C_49_H_65_N_4_O_5_^+^ I^-^).

LRMS: [ESI^+^, MeCN] *m/z*: 789.5 ([M]^+^, 100).

HRMS: [ESI^+^, MeCN] Calculated mass C_49_H_65_N_4_O_5_^+^ [M]^+^: 789.4950; found *m/z*: 789.4940.

^1^H NMR (500 MHz, CDCl_3_) δ 8.11 – 7.90 (m, 2 H, H-32, H-34), 7.42 – 7.32 (m, 4 H, H-5, H-5′, H-7, H-7′), 7.26 – 7.18 (m, 2 H, H-6, H-6′), 7.11 (d, *J* = 8.0 Hz, 1 H, H-8), 7.09 (d, *J* = 8.0 Hz, 1 H, H-8′), 7.02 (t, *J* = 12.5 Hz, 1 H, H-33), 6.80 (br. s, 1 H, H-16), 6.46 (d, *J* = 13.7 Hz, 1 H, H-31), 6.36 (d, *J* = 13.6 Hz, 1 H, H-35), 5.34 (br. s, 1 H, H-23), 4.13 (d, *J* = 8.0 Hz, 2 H, H-25), 4.09 (t, *J* = 7.3 Hz, 2 H, H-10), 3.66 (s, 3 H, H-37), 3.63 (s, 4 H, H-19, H-20), 3.59 (t, *J* = 5.6, 2 H, H-18), 3.57 (t, *J* = 5.4, 2 H, H-21), 3.46 (app q, *J* = 5.6 Hz, 2 H, H-17), 3.38 (app q, *J* = 5.4 Hz, 2 H, H-22), 2.34 (t, *J* = 7.3, 2 H, H-14), 2.29 – 2.16 (m, 6 H, H-29, H-28a), 1.89 – 1.80 (m, 2 H, H-11), 1.80 – 1.74 (m, 2 H, H-13), 1.74 (s, 6 H, H-36), 1.72 (s, 6 H, H-36′), 1.62 – 1.52 (m, 4 H, H-12, H-28b), 1.39 – 1.31 (m, 1 H, H-26), 0.97 – 0.86 (m, 2 H, H-27).

^13^C NMR (126 MHz, CDCl_3_) δ 172.3 (C2), 172.0 (C2′), 171.8 (C15), 155.9 (C24), 152.5 (C32), 151.9 (C34), 141.8 and 140.9 (C9, C9′), 140.2 and 139.8 (C4, C4′), 127.8 and 127.6 (C7, C7′), 125.8 (C33), 124.3 and 124.0 (C6, C6′), 121.2 and 121.1 (C5, C5′), 109.8 and 109.3 (C8, C8′), 103.4 (C31), 102.9 (C35), 97.8 (C30), 69.3, 69.2, 69.1 and 68.7 (C18, C19, C20, C21), 61.6 (C25), 48.4 and 48.1 (C3, C3′), 43.5 (C10), 39.8 (C22), 38.0 (C17), 35.2 (C14), 31.1 (C37), 28.0 (C28), 27.1 (2× C36, 2× C36′), 26.2 (C11), 25.5 (C12), 24.2 (C13), 20.4 (C29), 19.1 (C27), 16.8 (C26).

#### 5(6)-FAM-BCN

5(6)-FAM NHS ester (30 mg, 0.063 mmol, 1.0 eq, purchased from Sigma-Aldrich) was dissolved in anhydrous DCM/DMF (2.5 mL, 4:1, *v/v*), then anhydrous DIPEA (30 µL, 0.17 mmol, 2.7 eq) and BCN amine (31 mg, 0.096 mmol, 1.5 eq) were added. The reaction mixture was stirred at room temperature for 15 h, then the solvent was removed *in vacuo*. The residue was purified by column chromatography on silica (0% to 15% MeOH in DCM). 5(6)-FAM-BCN (27 mg, 0.040 mmol) was obtained in 63% yield as a yellow solid. M_w_ = 682.73 g/mol (Chemical Formula: C_38_H_38_N_2_O_10_). For ease of description 5(6)-FAM-BCN is written as FAM-BCN subsequently.

R_f_: 0.36 [DCM/MeOH 9:1 *v/v*]

LRMS: [ESI^+^, MeOH]: *m/z*: 683.1([M+H]^+^, 100); [ESI^-^, MeOH] *m/z*: 681.1 ([M-H]^-^, 100).

HRMS: [ESI^+^, MeOH] Calculated mass [M+H]^+^: 683.2600; found *m/z*: 683.2598.

^1^H NMR (500 MHz, CD_3_OD) δ 8.13 – 8.09 (m, 1 H, Ar-H), 8.04 (d, *J* = 8.0 Hz, 1 H, Ar-H), 7.61 (s, 1 H, Ar-H), 6.65 (d, *J* = 2.4 Hz, 2 H, H-2, H-2′), 6.59 (d, *J* = 8.8 Hz, 2 H, H-5, H-5′), 6.51 (dd, *J* = 8.8, 2.4 Hz, 2 H, H-4, H-4′), 4.03 (d, *J* = 8.0 Hz, 2 H, H-25), 3.55 (t, *J* = 5.5 Hz, 2 H, H-18), 3.54 – 3.49 (m, 4 H, H-19, H-20), 3.47 (t, *J* = 5.5 Hz, 2 H, H-17), 3.40 (t, *J* = 5.5 Hz, 2 H, H-21), 3.13 (t, *J* = 5.5 Hz, 2 H, H-22), 2.23 – 2.05 (m, 6 H, H-29, H-28a), 1.58 – 1.46 (m, 2 H, H-28b), 1.31 – 1.22 (m, 1 H, H-26), 0.91 – 0.80 (m, 2 H, H-27).

^13^C NMR (126 MHz, CD_3_OD) δ 169.2 (C10), 166.9 (C15), 160.6 (C1, C1′), 157.8 (C24), 152.8 (C3, C3′), 140.7 (C9), 129.0 (ArC, C5, C5′), 124.9 (ArC), 122.9 (ArC), 112.6 (C4, C4′), 109.7 (C6, C6′), 102.2 (C2, C2′), 98.1 (C30), 69.82 and 69.78 (C19, C20), 69.6 (C21), 68.9 (C18), 62.3 (C25), 40.2 (C22), 39.6 (C17), 28.7 (C28), 20.5 (C29), 20.0 (C27), 17.5 (C26).

# S2: General method for oligonucleotide synthesis and purification

Standard DNA phosphoramidites, solid supports, and additional reagents were purchased from Link Technologies or Applied Biosystems Ltd. All oligonucleotides were synthesised on an Applied Biosystems 394 automated DNA/RNA synthesiser using a standard 1.0 μmol scale phosphoramidite cycle of acid-catalysed detritylation, coupling, capping, and iodine oxidation. Stepwise coupling efficiencies and overall yields were determined by the automated trityl cation conductivity monitoring facility and in all cases were >98.0%. All β-cyanoethyl phosphoramidite monomers were dissolved in anhydrous acetonitrile to a concentration of 0.1 M immediately prior to use. The coupling time for normal A, G, C, and T monomers was 40 s, and this was extended to 600 s for 5′-FAM, 5′-phosphate and 5′-biotin phosphoramidites. Cleavage of oligonucleotides from the solid support and deprotection was achieved by exposure to concentrated aqueous ammonia solution for 60 min at room temperature followed by heating in a sealed tube for 5 h at 55 °C.

The building blocks for the RNA analogues were prepared using 2ʹ-*t*‑butyldimethylsilyl protected RNA phosphoramidite monomers with *t*-butylphenoxyacetyl protection of the A, G and C nucleobases and unprotected U (Sigma-Aldrich). A solution of 0.3 M benzylthiotetrazole in acetonitrile (Link Technologies) was used as the coupling agent, *t*-butylphenoxyacetic anhydride was employed as the capping agent and 0.1 M iodine as the oxidising agent (Sigma-Aldrich). All RNA phosphoramidite monomers were dissolved in anhydrous acetonitrile to a concentration of 0.1 M immediately prior to use, and the coupling time for all monomers was 10 min. Stepwise coupling efficiencies were determined by automated trityl cation conductivity monitoring and in all cases were >96%. Cleavage of oligonucleotides from the solid support and deprotection were achieved by exposure to concentrated aqueous ammonia/ethanol (3/1 *v/v*) for 2 h at room temperature followed by heating in a sealed tube for 1.5 h at 55 °C. After cleavage from the solid support and deprotection of the nucleobases and phosphotriesters, RNA oligonucleotides were concentrated to ~ 1 mL *in vacuo*, transferred to 15 mL plastic tubes and freeze dried. The residues were dissolved in DMSO (300 μL) and triethylamine trihydrofluoride (300 μL) was added after which the reaction mixtures were kept at 65 °C for 2.5 h. 3 M Sodium acetate (50 μL, pH 5.5) and butanol (3 mL) were added with vortexing and the samples were kept at -80 °C for 30 min then centrifuged at 4 °C at 15,000 × g for 10 min. The supernatant was decanted and the precipitate was washed twice with ethanol (0.75 mL) then dried under vacuum.

The fully deprotected DNA oligonucleotides were purified by reversed-phase HPLC on a Gilson system using a Luna 10u C8 100Å pore Phenomenex 10 × 250 mm column with a gradient of acetonitrile in ammonium acetate (buffer A: 0.1 M ammonium acetate, pH 7.0; buffer B: 0.1 M ammonium acetate, pH 7.0, with 50% acetonitrile; 3.5% to 35% buffer B over 20 min; flow rate 4 mL/min and monitored by UV absorption at 295 nm). After HPLC purification, oligonucleotides were desalted using NAP-10 columns (GE Healthcare). For HPLC purification of RNA, triethylammonium bicarbonate (TEAB) buffer was used (buffer A: 0.1 M TEAB, pH 7.5; buffer B: 0.1 M TEAB, pH 7.5, with 50% acetonitrile, 3.5% to 35% buffer B over 20 min). The fractions from HPLC were lyophilised, used without need for additional desalting and stored dry at -20 °C.

Table S1. Oligonucleotide sequences used in primer extension, reverse transcription and PCR reactions.

| Code | Sequence (5′-3′) | Mass  Found (Calc.) | |
| --- | --- | --- | --- |
| T1 | C**A**GTC**A**CTGT**A**CTGCCGACACACATAACC (DNA template) | 8775(8776) | |
| T2 | C**A**GTC**A**C**AAAA**CTGCCGACACACATAACC (DNA template) | 8777(8778) | |
| P3 | FAM-GGTTATGTGTGTCGGCAG (primer ) | 6138(6138) | |
| T4 | C**A**GUC**A**CUGU**A**CUGCCGACACACAUAACC (RNA template) | 9169(9169) | |
| T5 | C**A**GUC**A**C**AAAA**CUGCCGACACACAUAACC (RNA template) | 9199(9199) | |
| P6 | GCATTCGAGCAACGTAAG (PCR primer for T8) | 5532(5533) | |
| P7 | GGTTATGTGTGTCGGCAG (PCR primer for T8) | 5601(5602) | |
| P6_p_ | Phosphate-GCATTCGAGCAACGTAAG (T8 primer for λ-exonuclease digestion) | 5612(5612) | |
| P7_p_ | Phosphate-GGTTATGTGTGTCGGCAG (T8 primer for λ-exonuclease digestion) | 5681(5680) | |
| P6_b_ | Biotin-GCATTCGAGCAACGTAAG (T8 primer for streptavidin magnetic separation) | 5937(5937) | |
| T8 | GGTTATGTGTGTCGGCAGTATTGTCAGTGTGAATTCCAGAGTGTGAGATTGTGTGCTGGCGATCTTACGTTGCTCGAATGC (PCR template) | 25201(25199) | |
| P9 | GTTTGGCTTTAGAGGCTGGAG (PCR primer for T11) | 6548(6548) | |
| P10 | ACTGCAATACGAATAATGGCTAC (PCR primer for T11) | 7040(7041) | |
| P9_p_ | Phosphate-GTTTGGCTTTAGAGGCTGGAG (T11 primer for λ-exonuclease digestion) | 6627(6627) | |
| P10_p_ | Phosphate-ACTGCAATACGAATAATGGCTAC (T11 primer for λ-exonuclease digestion) | 7119(7120) | |
| T11 | plasmid HydGdCTD5 template (sequence in S6) |  | |
| FAM is 6-carboxamidohexylfluorescein. The RNA templates have the equivalent sequence to the DNA templates. The bold **A** shows the incorporation site of the modified triphosphates. | | |  |

# S3: Primer extension reactions

In a 20 µL reaction (1× buffer), 66 pmol of FAM labelled DNA primer (P3), 132 pmol of template (T1 or T2 in Table S1) and 3.2 nmol of AHP dUTP or unmodified dNTPs were mixed with polymerase enzyme (1 unit for Gotaq, Klenow and Therminator^TM^ II, 0.5 unit for KOD) and stock buffer solution (5× or 10× solution supplied with the enzymes). In the case of KOD polymerase, 1 mM MgCl_2_ was added separately to the reaction mixture. The reaction mixtures were heated for 1.5 h on a BIO-RAD T100^TM^ Thermal Cycler (Therminator^TM^ II, KOD and Gotaq at 60 or 72 °C, Klenow at 37 °C) and formamide (20 µL) was added before analysis by 20% denaturing polyacrylamide gel electrophoresis (PAGE) at a constant 20 W of power in 1× TBE buffer (pH 8.3). Gel images were taken using Syngene G:BOX with the gene-snap imaging software on a transilluminator (302 nm). For the mass spectrometry analysis, two identical reactions (2× 20 µL) were carried out, followed by ethanol precipitation. DNA was precipitated by mixing with 3 M sodium acetate (4 µL, pH 5.3) and ethanol (120 µL). The mixture was then left on dry ice for 10 min and at -20 °C for further 20 min. This was followed by centrifugation at 4 °C for 30 min (15,000 × g). The precipitate was dissolved in deionised water (15 µL) and analysed by HPLC-MS.

#### Primer extension reactions using AHP dUTP and template T1


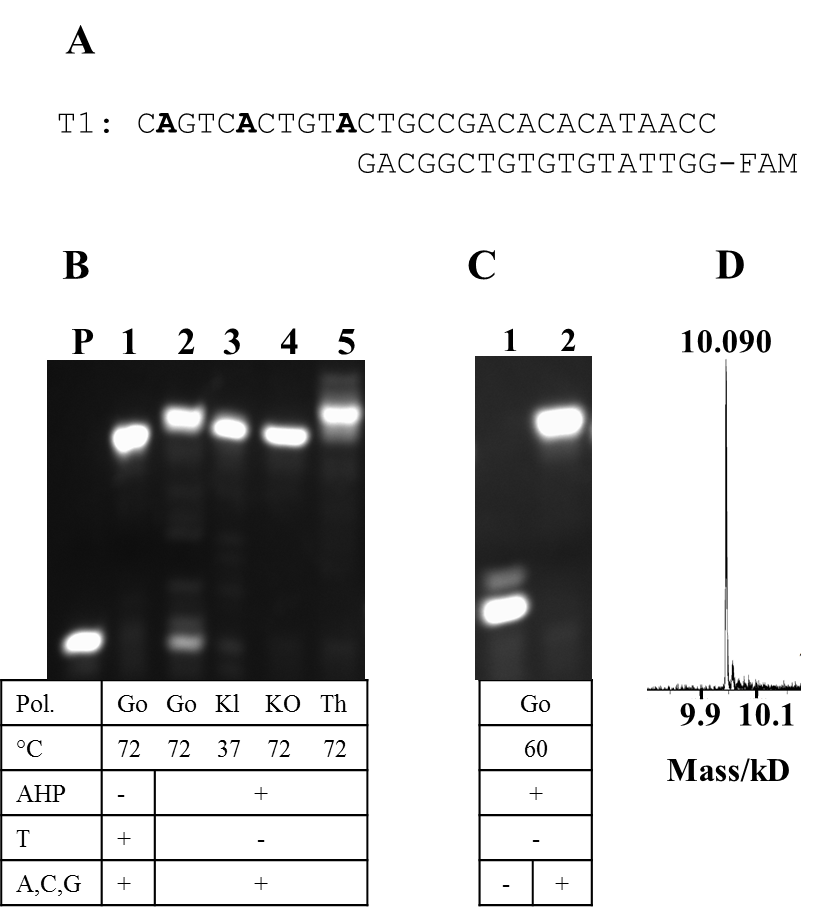


Figure S2. Primer extension using AHP dUTP (1.5 h). **A.** Template T1 and primer P3. **B.** 20% denaturing PAGE analysis of the reactions using Gotaq (Go), Klenow (Kl), KOD (KO) and Therminator II (Th) polymerases. Lane P, primer P3; lane 1, dNTPs; lanes 2 to 5, AHP dUTP + dATP + dCTP + dGTP. **C.** Gotaq reactions at 60 °C. Lane 1, AHP dUTP; lane 2, AHP dUTP + dATP + dCTP + dGTP. **D.** Mass spectrum of AHP dUTP fully extended product (calc. 10091) using Klenow.

#### Time course of primer extension reaction using Gotaq polymerase


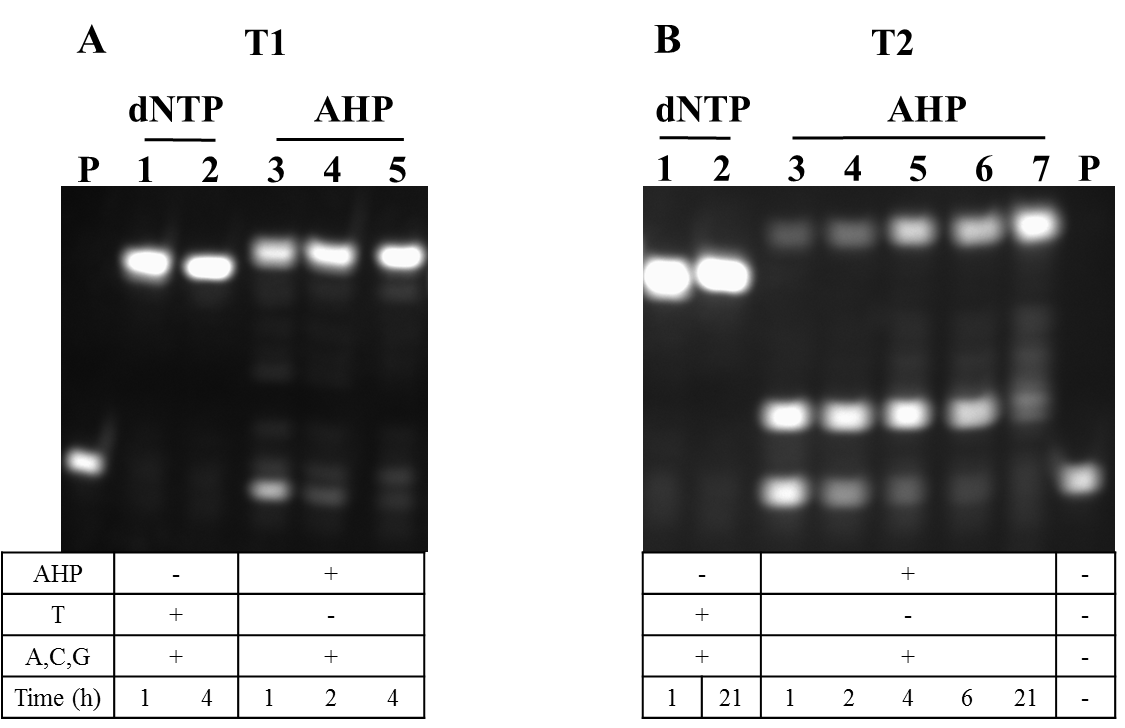


Figure S3. 20% denaturing PAGE analyses of the time course of primer extension reaction using AHP dUTP and Gotaq polymerase (72 °C). **A.** Reactions with primer P3 and template T1. Lane P, primer P3; Lanes 1 to 2, unmodified dNTP reactions for 1 and 4 h, respectively; Lanes 3 to 5, AHP dUTP + dATP + dCTP + dGTP reactions for 1, 2 and 4 h respectively. **B.** Reactions with primer P3 and template T2. Lane P, P3 + T2 without triphosphates; Lanes 1 to 2, unmodified dNTP reactions for 1 and 21 h, respectively; Lanes 3 to 7, AHP dUTP + dATP + dCTP + dGTP reactions for 1, 2, 4, 6 and 21 h respectively.

Denaturing PAGE and mass spectrometry (Table S3) analyses confirmed that T1-templated AHP dU products were successfully obtained using KOD, Gotaq, Therminator II and Klenow polymerases. Gotaq and Therminator II polymerases added one extra base to the fully extended products. Gotaq polymerase incorporated AHP dUTP into template T1 more efficiently at 60 °C than at 70 °C.

#### Kinetics of AHP dUTP incorporation

Primer extension reactions were prepared using above protocol with different concentrations of dTTP or AHP dUTP and heated at 60 °C on a BIO-RAD T100^TM^ Thermal Cycler. The reactions were quenched after 30, 60, 90, 120, 150, 180 and 210 s by freezing in liquid nitrogen and mixing with formamide/TBE buffer (20 μL). The reaction mixtures were analysed by 20% denaturing PAGE and quantified using gene-snap imaging software. The initial reaction rates were calculated from the yield of reaction divided as a function of reaction time. The data were fitted to Michaelis-Menten equation using Origin software to obtain the maximal reaction rate V_max_ and Michaelis constant K_m_. The supplier defined polymerase unit was used as polymerase concentration to calculate the relative catalytic constant K_cat_.

#### Kinetics of AHP dUTP incorporation using Gotaq polymerase


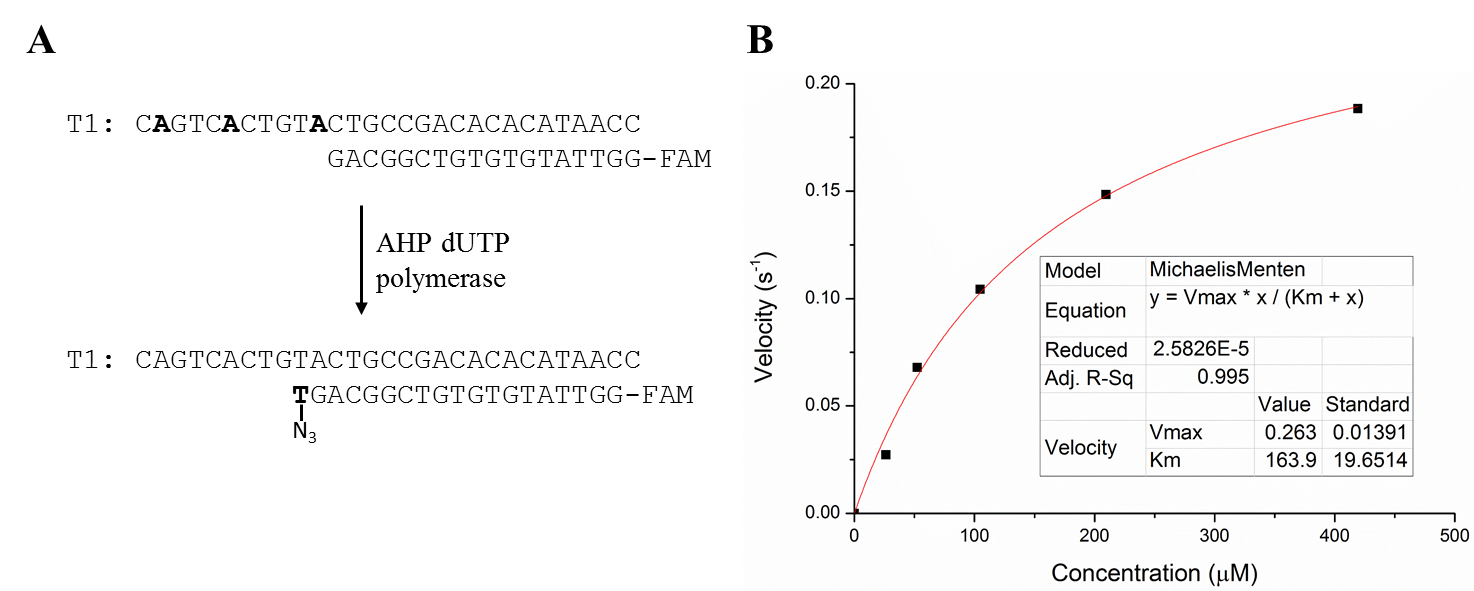


Figure S4. **A.** Single nucleotide incorporation during primer extension. **B.** The Michaelis-Menten fit for AHP dUTP using Gotaq polymerase.

Table S2. The kinetics of dTTP and AHP dUTP incorporation rate using Gotaq and KOD polymerases.

| Polymerase | dXTP | V_max_ | K_m_ | K_cat_ | K_cat_/K_m_ | |
| --- | --- | --- | --- | --- | --- | --- |
|  |  | (s^-1^) | (μM) | (s^-1^) | (s^-1^M^-1^) | Relative values |
| Gotaq | dTTP | 1.2 ± 0.08 | 61 ± 10 | 2.40 ± 0.16 | (3.9 ± 0.9) × 10^4^ | 1 |
|  | AHP dUTP | 0.26 ± 0.01 | 164 ± 20 | 0.52 ± 0.02 | (3.2 ± 0.5) × 10^3^ | 0.08 |
| KOD | dTTP | 0.33 ± 0.02 | 1.0 ± 0.2 | 0.66 ± 0.04 | (6.6 ± 1.8) × 10^5^ | 1 |
|  | AHP dUTP | 0.33 ± 0.01 | 1.1 ± 0.2 | 0.66 ± 0.02 | (6.0 ± 1.3) × 10^5^ | 0.91 |

Note: V_max_ is the maximal reaction rate; K_m_ is the Michaelis constant; K_cat_ is the catalytic constant; K_cat_/K_m_ is the catalytic efficiency.

#### Fluorescent labelling of primer extension products

Two 20 µL primer extension reactions were performed using azide dUTPs, or unmodified dNTPs as a negative control. Ethanol precipitation of the products was carried out as described in section S3 and the precipitates were re-dissolved in 1× Gotaq green buffer (40 µL). Half of the solution (20 µL) was reacted with 6.4 nmol each of Cy3-BCN, FAM-BCN or Cy5-BCN (2 mM stock solution of each dye in DMSO) at RT for the specific time. When the reaction was complete, another ethanol precipitation was performed. Formamide (20 µL) was added to the labelled and unlabelled products followed by analysis on a 20% denaturing PAGE at a constant 20 W of power. For the HPLC-MS analysis, two identical labelling reactions were carried out.


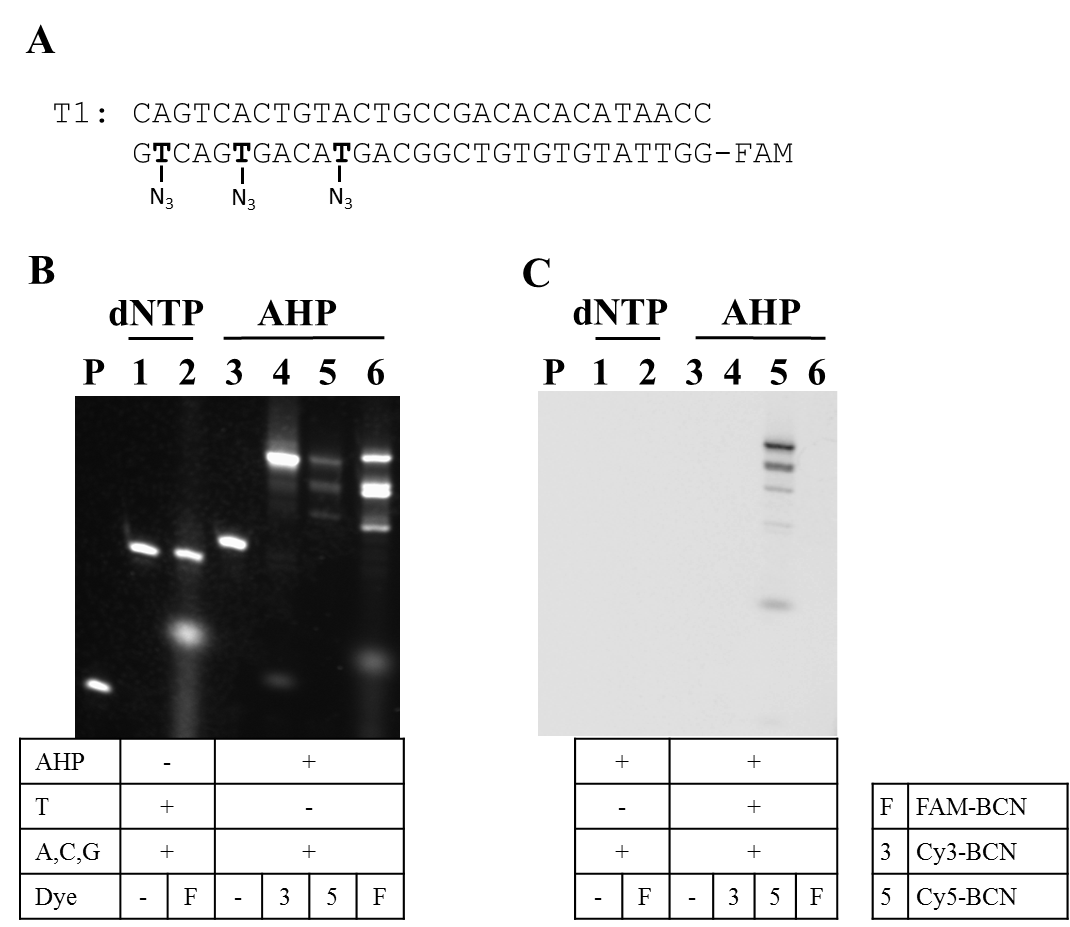


Figure S5. Fluorescent labelling of AHP-modified full-length products. **A.** AHP dU products from primer P3 and template T1. **B.** and **C.** 20% denaturing PAGE analysis visualised on a transilluminator and *via* Cy5 channel (for parameters, see Table S4). Lane P, primer P3; Lane 1, unmodified product; Lane 2, unmodified product mixed with FAM-BCN; Lane 3, AHP-modified product; Lanes 4 to 6, AHP-modified products labelled with Cy3-BCN ([5](#_ENREF_5)), Cy5-BCN and FAM-BCN individually at RT for 4 h.

The denaturing PAGE analysis demonstrates that labelling the AHP-modified full-length product with Cy3‑BCN is more efficient than FAM-BCN and Cy5-BCN.

# S4: Reverse transcription

In a 20 µL reaction (1× buffer), 66 pmol of FAM labelled DNA primer (P3), 132 pmol of RNA template (T4 and T5 in Table S1) and 3.2 nmol of AHP dUTP or unmodified dNTPs were mixed with reverse transcriptase (100 units for M-MuLV (RNase H^-^), or 5 units for AMV) and 10× buffer. 1× dithiothreitol (supplied with the enzyme) was added to the M-MuLV reactions, while 20 units of RNase inhibitor were added to the AMV reactions. The reaction mixtures were heated at 42 ºС for the specified time on a BIO-RAD T100^TM^ Thermal Cycler. When the reactions were complete, formamide (20 µL) was added and samples were analysed by 20% denaturing PAGE at a constant 20 W of power. For the mass spectrometry analysis, two identical reactions were carried out, followed by ethanol precipitation. The DNA precipitates were dissolved in deionised water (15 µL) and analysed by HPLC-MS.

#### Reverse transcription using AHP dUTP and template T4


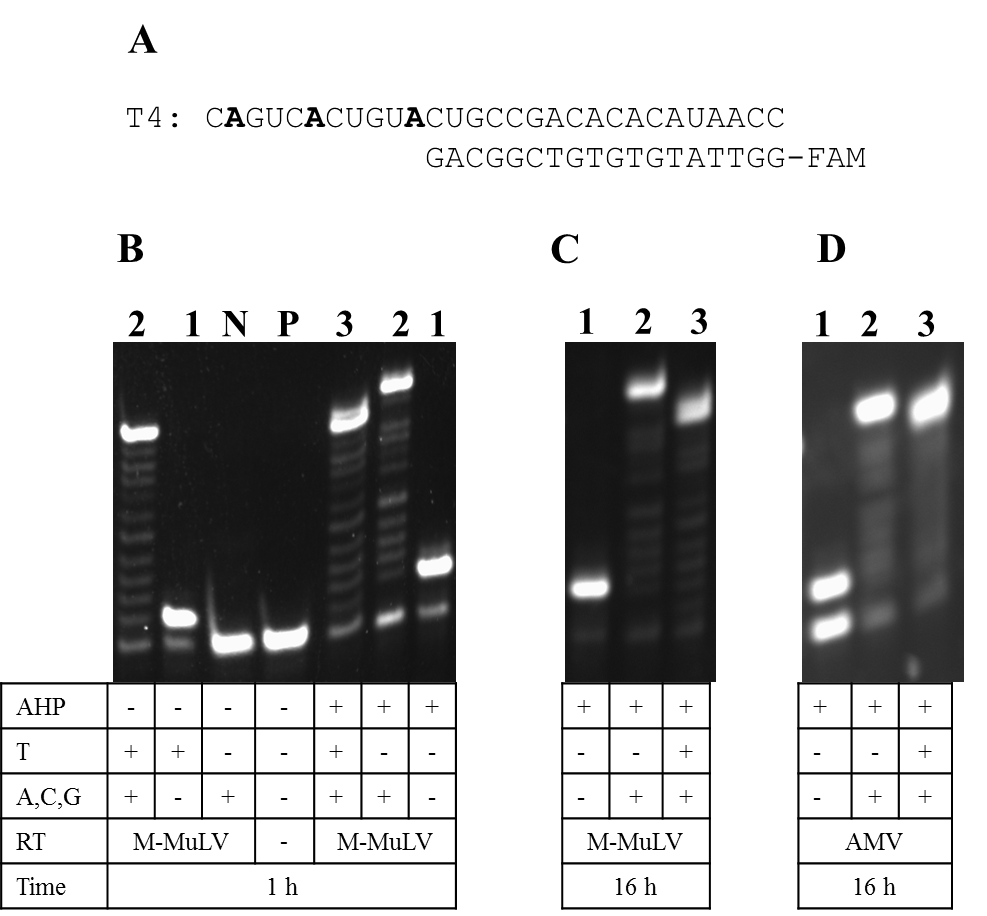


Figure S6. Reverse transcription using AHP dUTP. **A.** Primer P3 with RNA template T4. **B.** 20% denaturing PAGE analysis of reverse transcription using M-MuLV (RNase H^-^) reverse transcriptase for 1 h. **C.** Reactions using M-MuLV (RNase H^-^) reverse transcriptase for 16 h. **D.** Reactions using AMV reverse transcriptase for 16 h. 3.2 nmol of each triphosphate was used unless otherwise stated. Lane P, P3; Lane N, reactions with dATP + dCTP + dGTP; Lane 1, dTTP or AHP dUTP; Lane 2, dTTP or AHP dUTP + dATP + dCTP + dGTP; Lane 3, dTTP (1.6 nmol) + AHP dUTP (1.6 nmol) + dATP + dCTP + dGTP.

# S5: Mass spectrometry analysis of primer extension and reverse transcription products

Table S3. Mass spec data for primer extension and reverse transcription. A, C, G = dATP, dCTP and dGTP.

|  | **T1 or T4** | | | | **T2 or T5** | | | |
| --- | --- | --- | --- | --- | --- | --- | --- | --- |
|  | dXTP | | dXTP+A+C+G | | dXTP | | dXTP+A+G+C | |
|  | Calc. mass | Found | Calc. mass | Found | Calc. mass | Found | Calc. mass | Found |
| dTTP^all^ | 6442 | 6442 | 9556 | 9555 | 7355 | 7355 | 9553 | 9553 |
| AHP | 6620 | 6621^Q,D,K,M^ | 10090 | 10090^Q,D,K,M^ | 8067 | 8067^all^ | 10621 | 10621^all^ |

Note: The superscripts indicate the specific polymerase for which the product was observed. All = all five enzymes (Q = Gotaq, K = Klenow, D = KOD, Therminator II, M = M-MuLV (RNase H)).

# S6: PCR amplification

#### PCR Protocol

For template T8, Gotaq polymerase (1 unit), 5× Gotaq green buffer (4 µL) and SYBR Green (0.6 µL, 3.75×) were added to the mixture containing 10 pmol of two primers (P6, P7), 50 pg of template (T8), 5 nmol in total of AHP dUTP/dTTP in addition to 5 nmol each of dATP, dCTP and dGTP. The final reaction volume was 20 µL with 1× reaction buffer. KOD polymerase (0.25 unit) was also used with an additional 1 mM MgCl_2_. For plasmid template T11, KOD polymerase (0.5 unit), 10× KOD polymerase buffer 1 (2 µL, pH 8.0), 25 mM MgCl_2_ (0.8 µL) and SYBR Green (0.6 µL, 3.75×) were added to the mixture containing 10 pmol of the two primers (P9, P10), 300 pg of plasmid template (T11), 10 nmol in total of AHP dUTP/dTTP in addition to 10 nmol each of dATP, dCTP and dGTP. The final reaction volume was 20 µL with 1× reaction buffer and 1 mM MgCl_2_.

Amplification was performed on a BIO-RAD CFX96 real-time PCR instrument using the following procedure: an initial denaturing at 95 ºC for 2 min, followed by 25 cycles of denaturing at 95 ºC for 30 s, annealing at 51 ºC (T8) or 54 ºC (T11) for 30 s, and elongating at 72 ºC for 30 s. Further extension was carried out at 72 ºC for 5 min. This was followed by melting temperature measurements involving heating the reaction mixture to 95 °C for 30 s, cooling to 30 °C, then increasing the temperature to 95 °C at a rate of 1 or 0.5 °C/s and holding (monitoring) at each temperature for 5 s. Samples were analysed by 1.5% or 2% agarose gel electrophoresis with ethidium bromide (200 ng/mL) at constant voltage (126 V) in 1× TBE buffer.

When the same PCR amplifications were repeated 5 to 10 times, errors of melting temperatures were calculated using the confidence intervals derived from Student’s *t* distribution with 95% confidence. In the individual graphs these errors are represented by error bars.

#### PCR using template T8


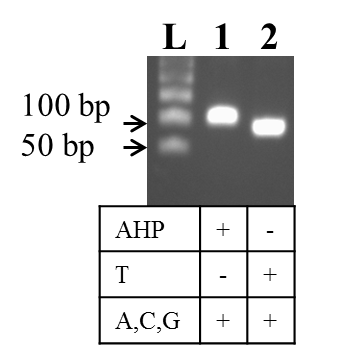


Figure S7. 2% agarose gel analysis of PCR amplification using template T8 and Gotaq polymerase. Lane L, 50 bp ladder; Lane 1, AHP dUTP + dATP + dCTP + dGTP; Lane 2, dNTPs.

# S7: Sequencing of PCR products (T11)

#### Amplification region of plasmid HydGdCTD5 template (T11)

501 AAGATATGGG ACATAAACGT TTGGCTTTAG AGGCTGGAGA AGACCCTGTA

551 AACAATCCTA TTGAATATAT TCTTGACTGT ATCAAAACCA TATACAGCAT

601 AAAACATAAA AATGGAGCAA TTAGACGTGT AAATGTAAAT ATTGCAGCTA

651 CTACTGTAGA AAACTACAAG AAATTAAAGG ATGCTGGTAT TGGAACATAT

701 ATACTTTTCC AAGAAACCTA TAACAAAAAA AGTTACGAGG AACTTCATCC

751 TACAGGTCCA AAACATGATT ATGCCTATCA TACAGAAGCA ATGGATCGTG

801 CTATGGAAGG TGGTATTGAT GATGTAGGTA TTGGGGTTTT GTTTGGACTA

851 AATATGTACA AATATGACTT TGTTGGACTT CTAATGCATG CTGAACACTT

901 GGAAGCTGCT ATGGGTGTAG GCCCTCATAC TATAAGCGTT CCTCGTATAC

951 GTCCTGCAGA TGACATTGAT CCTGAAAACT TCTCAAATGC AATATCGGAC

1001 GAGATTTTTG AAAAAATTGT AGCCATTATT CGTATTGCAG TTCCATACAC

Primer 9 GTTTGGCTTTAGAGGCTGGAG

Primer 10 ACTGCAATACGAATAATGGCTAC

PCR amplifications using AHP dUTP and T11 were carried out as previously described and purified by agarose gel electrophoresis. Products were extracted using a Qiagen gel extraction kit according to the manufacturer’s instructions. The solution of AHP‑modified PCR amplicons were quantified using a NanoDrop 2000 instrument. Additionally, second-round PCR reactions were carried out using unmodified triphosphates, Gotaq polymerase and the AHP‑modified PCR products (10 ng) as templates. The products were purified using agarose gel electrophoresis (1.5%), followed by extraction using a Qiagen gel extraction kit. The AHP‑modified PCR amplicons and second‑round PCR products were then despatched to Beckman Coulter Genomics Inc. for Sanger sequencing.

Sequencing of the AHP-modified PCR products terminated early in proportion to the ratios of AHP-dUTP to TTP used in the PCR reaction: (281 bp (100% AHP dU), 173 bp (75% AHP dU), 351 bp (50% AHP dU), full length amplicon (25% AHP dU)).

#### Second-round PCR using AHP-modified templates


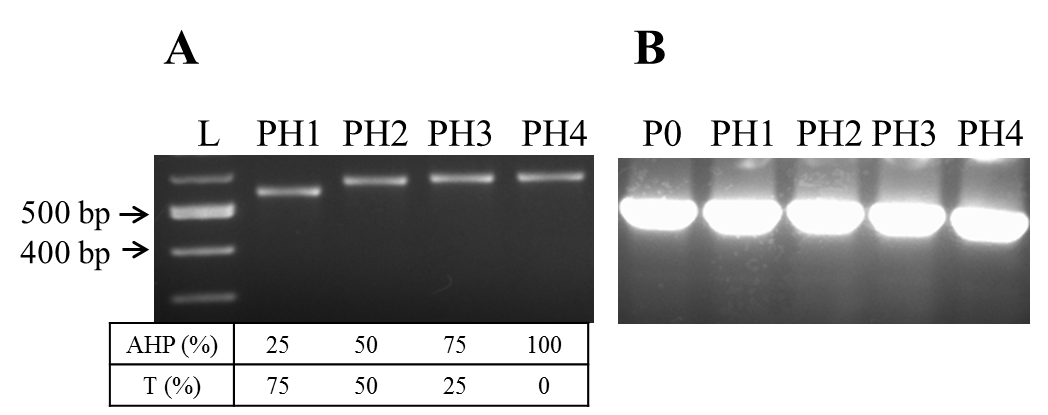


Figure S8. Second-round PCR amplification using AHP-modified products as templates. **A.** 1.5% agarose gel analysis of the AHP-modified templates (extracted as explained above, 50 ng each). **B.** Amplicons from second-round PCR using modified templates, dNTPs and Gotaq polymerase. Lane L, 100 bp ladder; Lane P0, unmodified amplicon template; Lane PH1 to PH4, 25% to 100% AHP-modified templates.

#### Sequencing results

Sequencing results of the second-round PCR products (523 bp) from the AHP-modified templates. The yellow and grey regions are the two primers. P0, amplicon from unmodified template; PH1 to PH4, amplicons from 25% to 100% AHP-modified templates; template, the original template T11.

Template starts: ACTGCAATACGAATAATGGCTACAATTTTTTCAAAAATCTCGTCCGATATTGCATTT

10 20 30 40 50

P0_primer_10 GAGAAGTTTT CAGGATCAAT GTCATCTGCA GGACGTATAC GAGGAACGCT

PH1_primer_10 GAGAAGTTTT CAGGATCAAT GTCATCTGCA GGACGTATAC GAGGAACGCT

PH2_primer_10 GAGAAGTTTT CAGGATCAAT GTCATCTGCA GGACGTATAC GAGGAACGCT

PH3_primer_10 GAGAAGTTTT CAGGATCAAT GTCATCTGCA GGACGTATAC GAGGAACGCT

PH4_primer_10 GAGAAGTTTT CAGGATCAAT GTCATCTGCA GGACGTATAC GAGGAACGCT

Template GAGAAGTTTT CAGGATCAAT GTCATCTGCA GGACGTATAC GAGGAACGCT

60 70 80 90 100

P0_primer_10 TATAGTATGA GGGCCTACAC CCATAGCAGC TTCCAAGTGT TCAGCATGCA

PH1_primer_10 TATAGTATGA GGGCCTACAC CCATAGCAGC TTCCAAGTGT TCAGCATGCA

PH2_primer_10 TATAGTATGA GGGCCTACAC CCATAGCAGC TTCCAAGTGT TCAGCATGCA

PH3_primer_10 TATAGTATGA GGGCCTACAC CCATAGCAGC TTCCAAGTGT TCAGCATGCA

PH4_primer_10 TATAGTATGA GGGCCTACAC CCATAGCAGC TTCCAAGTGT TCAGCATGCA

Template TATAGTATGA GGGCCTACAC CCATAGCAGC TTCCAAGTGT TCAGCATGCA

110 120 130 140 150

P0_primer_10 TTAGAAGTCC AACAAAGTCA TATTTGTACA TATTTAGTCC AAACAAAACC

PH1_primer_10 TTAGAAGTCC AACAAAGTCA TATTTGTACA TATTTAGTCC AAACAAAACC

PH2_primer_10 TTAGAAGTCC AACAAAGTCA TATTTGTACA TATTTAGTCC AAACAAAACC

PH3_primer_10 TTAGAAGTCC AACAAAGTCA TATTTGTACA TATTTAGTCC AAACAAAACC

PH4_primer_10 TTAGAAGTCC AACAAAGTCA TATTTGTACA TATTTAGTCC AAACAAAACC

Template TTAGAAGTCC AACAAAGTCA TATTTGTACA TATTTAGTCC AAACAAAACC

160 170 180 190 200

P0_primer_10 CCAATACCTA CATCATCAAT ACCACCTTCC ATAGCACGAT CCATTGCTTC

PH1_primer_10 CCAATACCTA CATCATCAAT ACCACCTTCC ATAGCACGAT CCATTGCTTC

PH2_primer_10 CCAATACCTA CATCATCAAT ACCACCTTCC ATAGCACGAT CCATTGCTTC

PH3_primer_10 CCAATACCTA CATCATCAAT ACCACCTTCC ATAGCACGAT CCATTGCTTC

PH4_primer_10 CCAATACCTA CATCATCAAT ACCACCTTCC ATAGCACGAT CCATTGCTTC

Template CCAATACCTA CATCATCAAT ACCACCTTCC ATAGCACGAT CCATTGCTTC

210 220 230 240 250

P0_primer_10 TGTATGATAG GCATAATCAT GTTTTGGACC TGTAGGATGA AGTTCCTCGT

PH1_primer_10 TGTATGATAG GCATAATCAT GTTTTGGACC TGTAGGATGA AGTTCCTCGT

PH2_primer_10 TGTATGATAG GCATAATCAT GTTTTGGACC TGTAGGATGA AGTTCCTCGT

PH3_primer_10 TGTATGATAG GCATAATCAT GTTTTGGACC TGTAGGATGA AGTTCCTCGT

PH4_primer_10 TGTATGATAG GCATAATCAT GTTTTGGACC TGTAGGATGA AGTTCCTCGT

Template TGTATGATAG GCATAATCAT GTTTTGGACC TGTAGGATGA AGTTCCTCGT

260 270 280 290 300

P0_primer_10 AACTTTTTTT GTTATAGGTT TCTTGGAAAA GTATATATGT TCCAATACCA

PH1_primer_10 AACTTTTTTT GTTATAGGTT TCTTGGAAAA GTATATATGT TCCAATACCA

PH2_primer_10 AACTTTTTTT GTTATAGGTT TCTTGGAAAA GTATATATGT TCCAATACCA

PH3_primer_10 AACTTTTTTT GTTATAGGTT TCTTGGAAAA GTATATATGT TCCAATACCA

PH4_primer_10 AACTTTTTTT GTTATAGGTT TCTTGGAAAA GTATATATGT TCCAATACCA

Template AACTTTTTTT GTTATAGGTT TCTTGGAAAA GTATATATGT TCCAATACCA

310 320 330 340 350

P0_primer_10 GCATCCTTTA ATTTCTTGTA GTTTTCTACA GTAGTAGCTG CAATATTTAC

PH1_primer_10 GCATCCTTTA ATTTCTTGTA GTTTTCTACA GTAGTAGCTG CAATATTTAC

PH2_primer_10 GCATCCTTTA ATTTCTTGTA GTTTTCTACA GTAGTAGCTG CAATATTTAC

PH3_primer_10 GCATCCTTTA ATTTCTTGTA GTTTTCTACA GTAGTAGCTG CAATATTTAC

PH4_primer_10 GCATCCTTTA ATTTCTTGTA GTTTTCTACA GTAGTAGCTG CAATATTTAC

Template GCATCCTTTA ATTTCTTGTA GTTTTCTACA GTAGTAGCTG CAATATTTAC

360 370 380 390 400

P0_primer_10 ATTTACACGT CTAATTGCTC CATTTTTATG TTTTATGCTG TATATGGTTT

PH1_primer_10 ATTTACACGT CTAATTGCTC CATTTTTATG TTTTATGCTG TATATGGTTT

PH2_primer_10 ATTTACACGT CTAATTGCTC CATTTTTATG TTTTATGCTG TATATGGTTT

PH3_primer_10 ATTTACACGT CTAATTGCTC CATTTTTATG TTTTATGCTG TATATGGTTT

PH4_primer_10 ATTTACACGT CTAATTGCTC CATTTTTATG TTTTATGCTG TATATGGTTT

Template ATTTACACGT CTAATTGCTC CATTTTTATG TTTTATGCTG TATATGGTTT

410 420 430 440 450

P0_primer_10 TGATACAGTC AAGAATATAT TCAATAGGAT TGTTTACAGG GTCTTCTCCA

PH1_primer_10 TGATACAGTC AAGAATATAT TCAATAGGAT TGTTTACAGG GTCTTCTCCA

PH2_primer_10 TGATACAGTC AAGAATATAT TCAATAGGAT TGTTTACAGG GTCTTCTCCA

PH3_primer_10 TGATACAGTC AAGAATATAT TCAATAGGAT TGTTTACAGG GTCTTCTCCA

PH4_primer_10 TGATACAGTC AAGAATATAT TCAATAGGAT TGTTTACAGG GTCTTCTCCA

Template TGATACAGTC AAGAATATAT TCAATAGGAT TGTTTACAGG GTCTTCTCCA

456

P0_primer_10 GCCTCA

PH1_primer_10 GCCTCA

PH2_primer_10 GCCTCA

PH3_primer_10 GCCTCA

PH4_primer_10 GCCTCA

Template GCCTCTAAAGCCAAAC

# S8: Synthesis of fluorescent double-stranded probes (T11)

Studies of labelling reaction buffer and time course were carried out and demonstrated that reaction in Gotaq green buffer between 30 to 60 min afforded intense labelled and aqueous soluble products.

#### Cy3-BCN labelled double-stranded probes (T11)


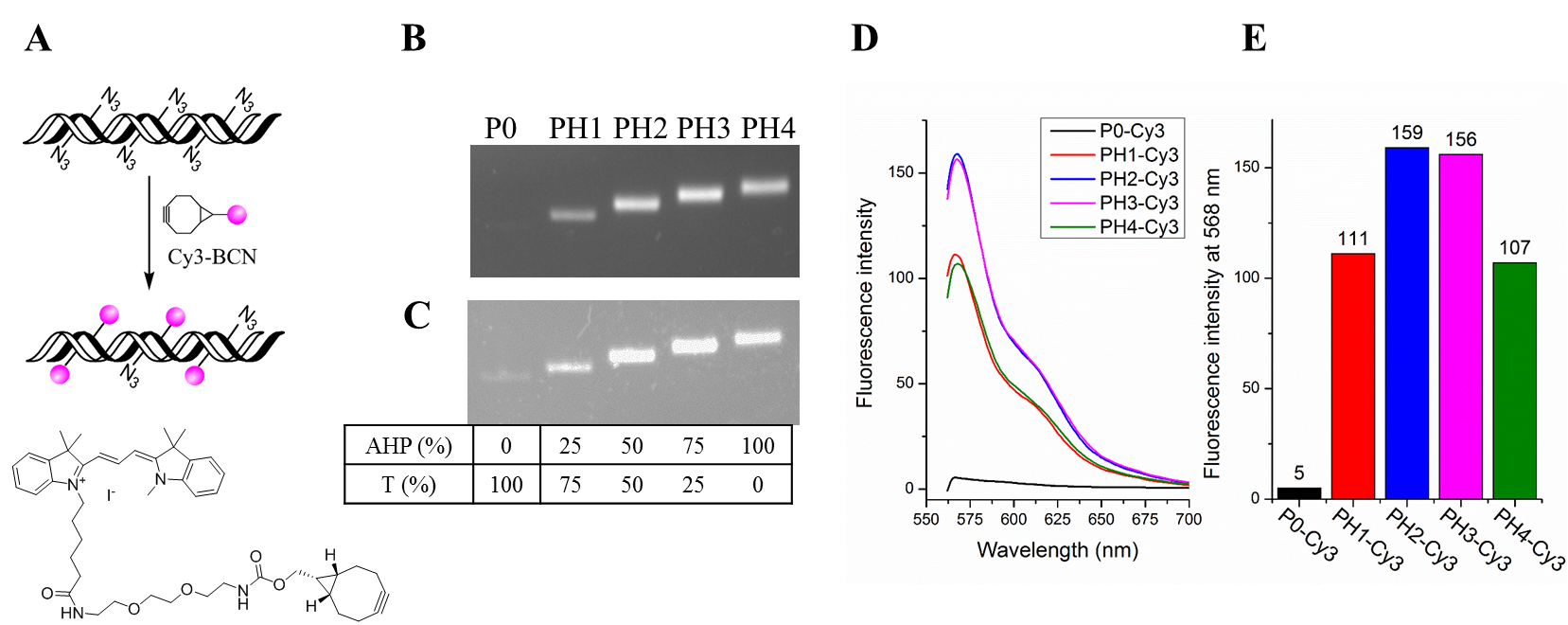


Figure S9. Cy3-BCN fluorescent labelling of AHP-modified PCR amplicons. **A.** Scheme for Cy3‑BCN labelling. **B.** and **C.** 1.5% agarose gel analysis of labelling reactions before staining and after staining with ethidium bromide. **D.** Fluorescence spectra of labelled PCR amplicons in deionised water, excited at 545 nm and recorded on a fluorimeter (Perkin Elmer LS50B Luminescence Spectrometer). **E.** The fluorescence intensity (relative fluorescence intensity, RFU) at 568 nm for each sample. P0, unmodified amplicon mixed with Cy3-BCN, negative control; PH1 to PH4, 25% to 100% AHP-modified PCR amplicons labelled with Cy3-BCN at RT for 30 min.

#### Cy5-BCN labelled double-stranded probes (T11)

Cy5 is more hydrophobic than FAM and Cy3, so the aqueous solubility of the labelled PCR product decreases. Instead of using gel extraction to purify the Cy5-BCN labelled products, excess free dye was removed using ethanol precipitation. The DNA precipitates were re-dissolved in deionised water and fluorescence was recorded on the fluorimeter.


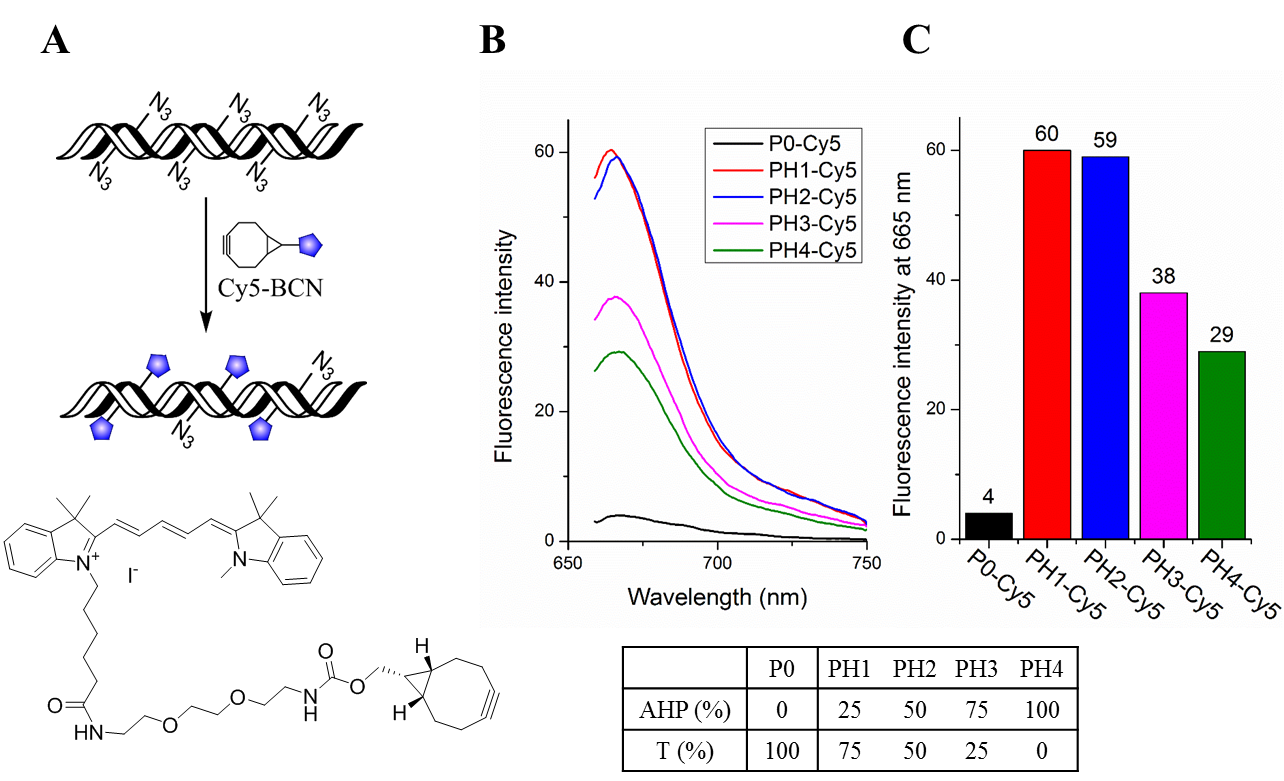


Figure S10. Cy5-BCN fluorescent labelling of AHP-modified PCR amplicons. **A.** Scheme for Cy5‑BCN labelling. **B.** Fluorescence spectra of labelled PCR amplicons in deionised water, excited at 645 nm and recorded on a fluorimeter. **C.** The fluorescence intensity (RFU) at 665 nm for each sample. P0, unmodified amplicon mixed with Cy5‑BCN, negative control; PH1 to PH4, 25% to 100% AHP-modified PCR amplicons labelled with Cy5-BCN at RT for 30 min.

# S9: Synthesis of fluorescent single-stranded probes

#### Exonuclease digestion protocol

PCR was carried out with one unmodified primer and one 5′-phosphorylated primer using different ratios of AHP dUTP to dTTP. In the asymmetric reaction (APCR), the ratio of unmodified primer to phosphorylated-primer was 10:1. The PCR/APCR protocol was the same as described in the PCR protocol (section S6), except the APCR reaction cycles were increased in number to 30. The reaction mixtures were washed with phenol/chloroform/isoamyl alcohol (25:24:1, saturated with Tris-HCl, Invitrogen) and precipitated with ethanol as explained section S3 to remove the DNA polymerase. The DNA precipitates were re-dissolved in 1× λ-exonuclease buffer (20 μL) containing 5 units (T8 products) or 10 units (T11 products) of λ-exonuclease and SYBR green (0.6 μL, 3.75×). The reactions were heated at 37 °C for 2 h on a BIORAD CFX96 real-time PCR instrument and this was followed by fluorescence melting analysis to check for the presence of remaining duplex products. When un-digested duplexes were observed, an extra 5 units of λ‑exonuclease was added and the reaction was heated at 37 °C for 1 h. When the digestion was complete, the single-stranded DNA in solution (10 μL) was mixed with the complementary strand and melting experiments were carried out. The single-stranded and re‑annealed samples were analysed on a 1.5% or 2% agarose gel with ethidium bromide or SYBR gold (0.5×) at constant voltage (126 V) in 1× TBE buffer.

#### Exonuclease digestion of AHP-modified APCR products (T11)


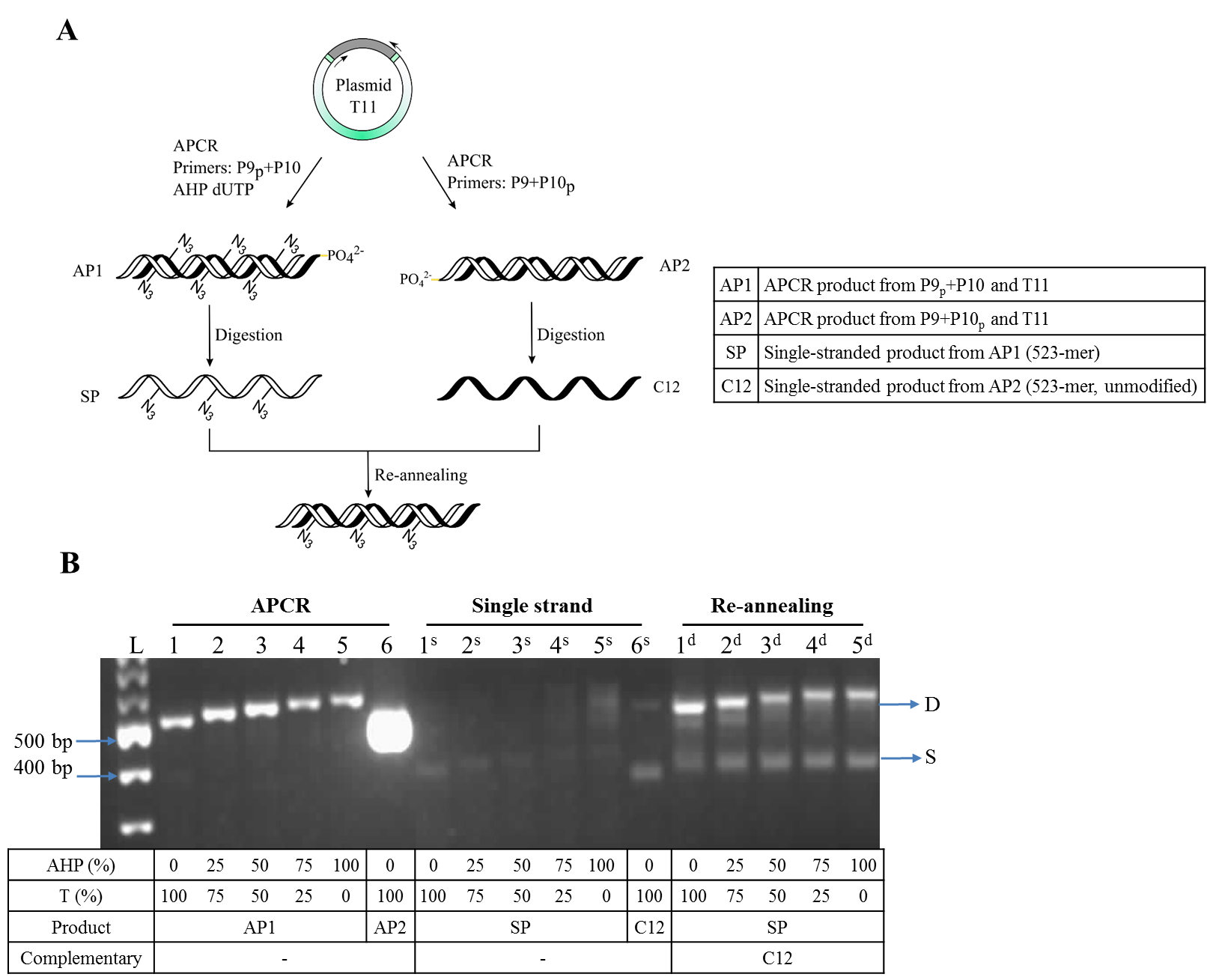


Figure S11. λ-exonuclease digestion of APCR amplicons (T11) and re-annealing of the AHP-modified single strands (SP) with the complementary unmodified strand C12. **A.** Scheme for APCR amplicon digestion and re-annealing. **B.** 1.5% agarose gel analysis containing ethidium bromide. Lane L, 100 bp ladder; lanes 1/1^s^/1^d^ and 6/6^s^, unmodified products; lanes 2/2^s^/2^d^ to 5/5^s^/5^d^, 25% to 100% AHP-modified products. D = duplex products; S = single strands.

The APCR reaction with primer P9 (T_m_ = 54.3 °C) as the limited primer was amplified more efficient than those with primer P10 (T_m_ = 52.8 °C) due to the higher T_m_ of the P9 duplex. ([6](#_ENREF_6),[7](#_ENREF_7)) Single-stranded products were obtained after λ-exonuclease digestion. The duplex bands were clearly observed after annealing, suggesting the presence of the single-stranded products.

#### FAM-BCN labelled single-stranded probes (T8)


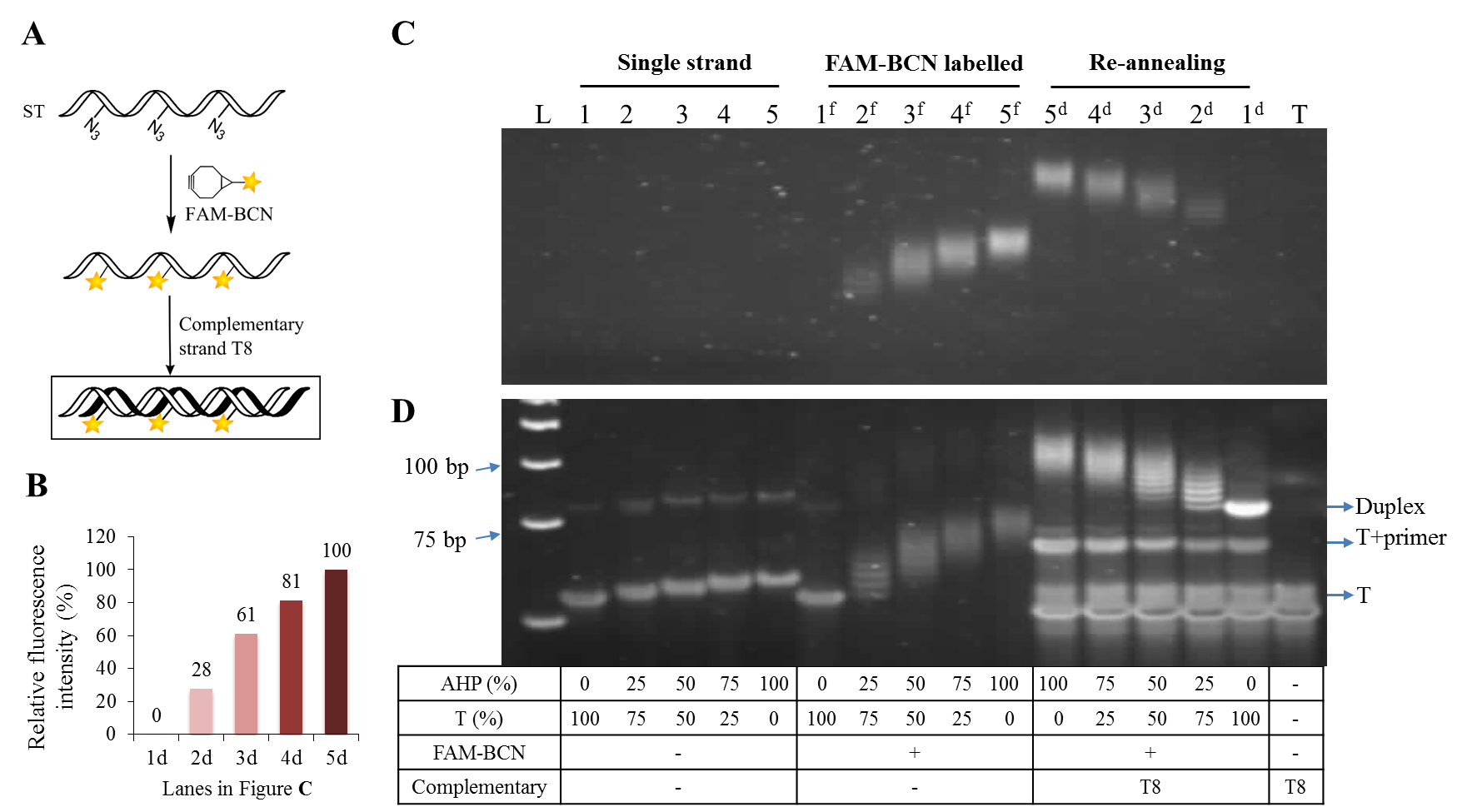


Figure S12. FAM-BCN fluorescent labelling of AHP-modified single strands (81-mer) and re-annealing of the labelled products with template T8. **A.** Scheme for fluorescent labelling and re-annealing. **B.** Relative fluorescence intensity for the re-annealed duplex bands quantified by gel analysis (lanes 1^d^ to 5^d^ in **C**, quantified using ImageJ). **C.** and **D.** 12% native PAGE analysis before staining and after staining with SYBR Gold. Lane L, 25 bp ladder; lane 1/1^f^/1^d^, unmodified product, negative control; lanes 2/2^f^/2^d^ to 5/5^f^/5^d^, 25% to 100% AHP‑modified products; lane T, template T8. Duplex = duplex products; T + primer, excess primer annealed to template T8; T = template T8.

The gels show that DNA single strands with AHP dU modifications were efficiently labelled when treated with FAM-BCN. The labelled single-stranded products can also be re-annealed to the complementary strand.

#### FAM-BCN labelled single-stranded probes (T11)


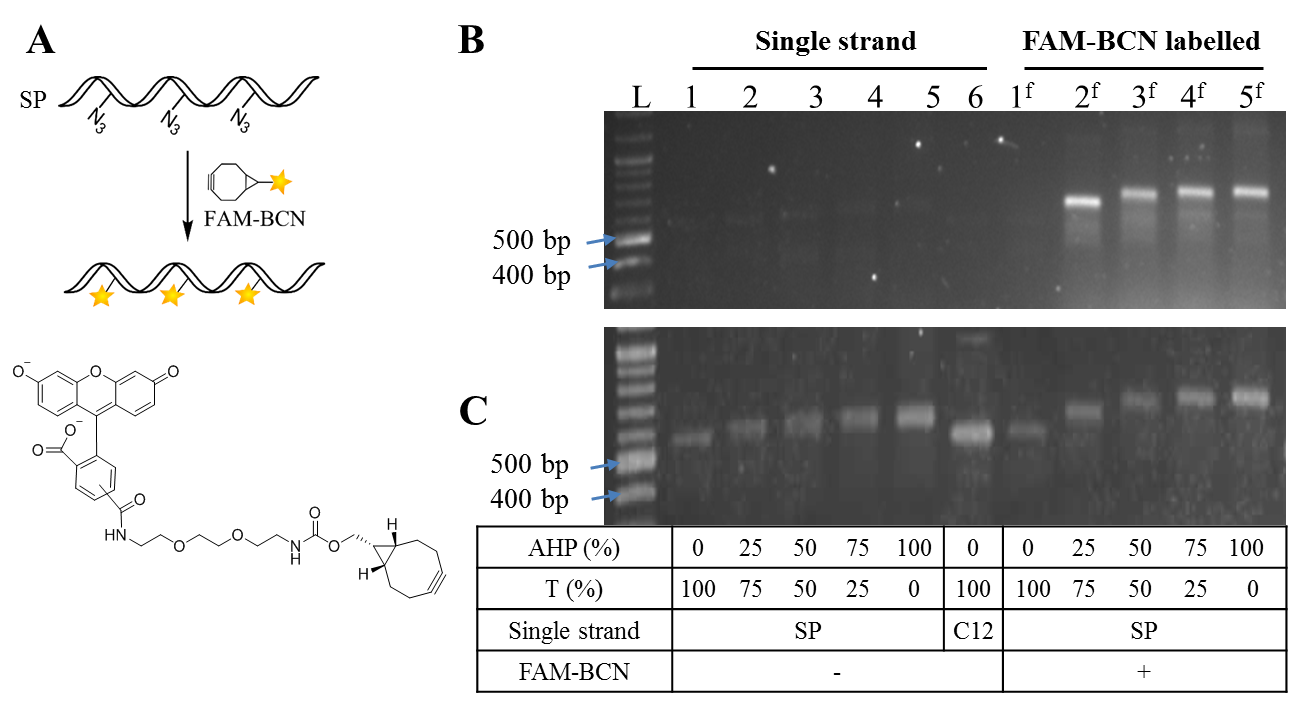


Figure S13. FAM-BCN fluorescent labelling of AHP-modified single strands (523-mer). **A.** Scheme for fluorescent labelling. **B.** and **C.** 1.5% agarose gel analysis before staining and after staining with ethidium bromide. Lane L, 100 bp ladder; lanes 1/1^f^ and 6, unmodified products, controls; lanes 2/2^f^ to 5/5^f^, 25% to 100% AHP-modified products.

#### FAM-BCN and Rhodamine-B-BCN dual-labelled single-stranded probes (T8)


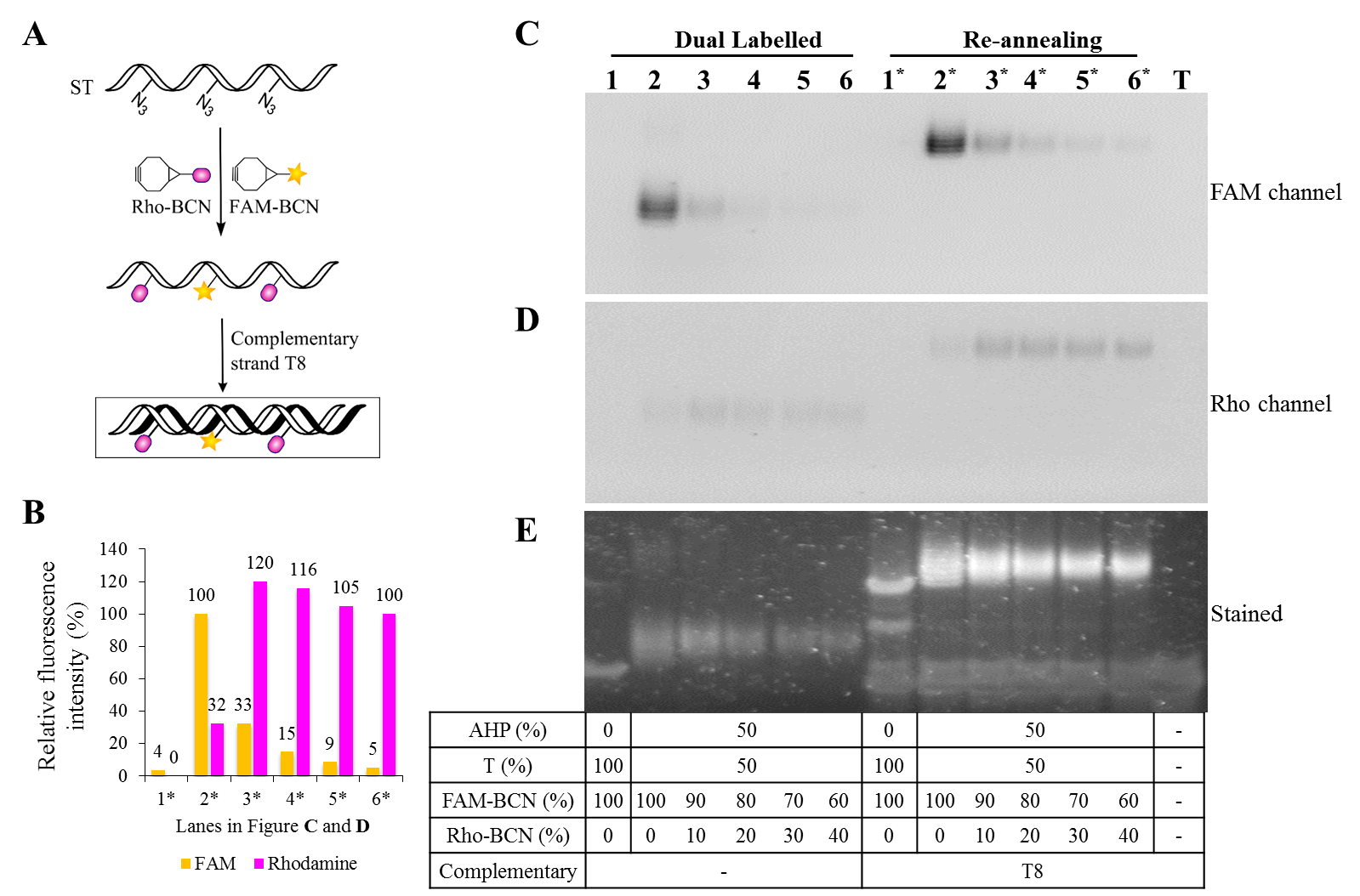


Figure S14. Dual labelling of 50% AHP-modified single strands (ST, 81-mer) with different combinations of FAM-BCN (100% to 60%) and Rhodamine-B-BCN (0% to 40%). **A.** Scheme for dual labelling and re-annealing. **B.** Relative fluorescence intensity for the re‑annealed duplex products quantified by gel analysis in FAM and Rhodamine channels (lanes 1^*^ to 6^*^ in **C** and **D**, quantified using ImageJ). **C.** **D.** and **E.** 12% native PAGE analysis visualised before staining in FAM channel, Rhodamine channel and after staining with SYBR Gold respectively. Lane 1/1^*^, unmodified product, negative control; lanes 2/2^*^ to 6/6^*^, 50% AHP‑modified products.

Table S4. Gel imaging parameters for dual-labelling system

|  | Light | Filter |
| --- | --- | --- |
| Stained gel | Transilluminator (302 nm) | EtBr/UV (572 - 625 nm) |
| FAM Channel | Epi-blue (455 - 487 nm) | Short wavelength (516 - 599 nm) |
| Cy3 (Rhodamine) Channel | Epi-green (520 - 550 nm) | Long wavelength (611 - 641 nm) |
| Cy5 Channel | Epi-red (615 - 650 nm) | FRLP (670 - 780 nm) |

Note: Pictures from FAM, Cy3 (Rhodamine) and Cy5 channels are shown as negative grey scale images.

#### FAM-BCN and Cy3-BCN dual labelling of ssDNA (T8) on streptavidin magnetic beads


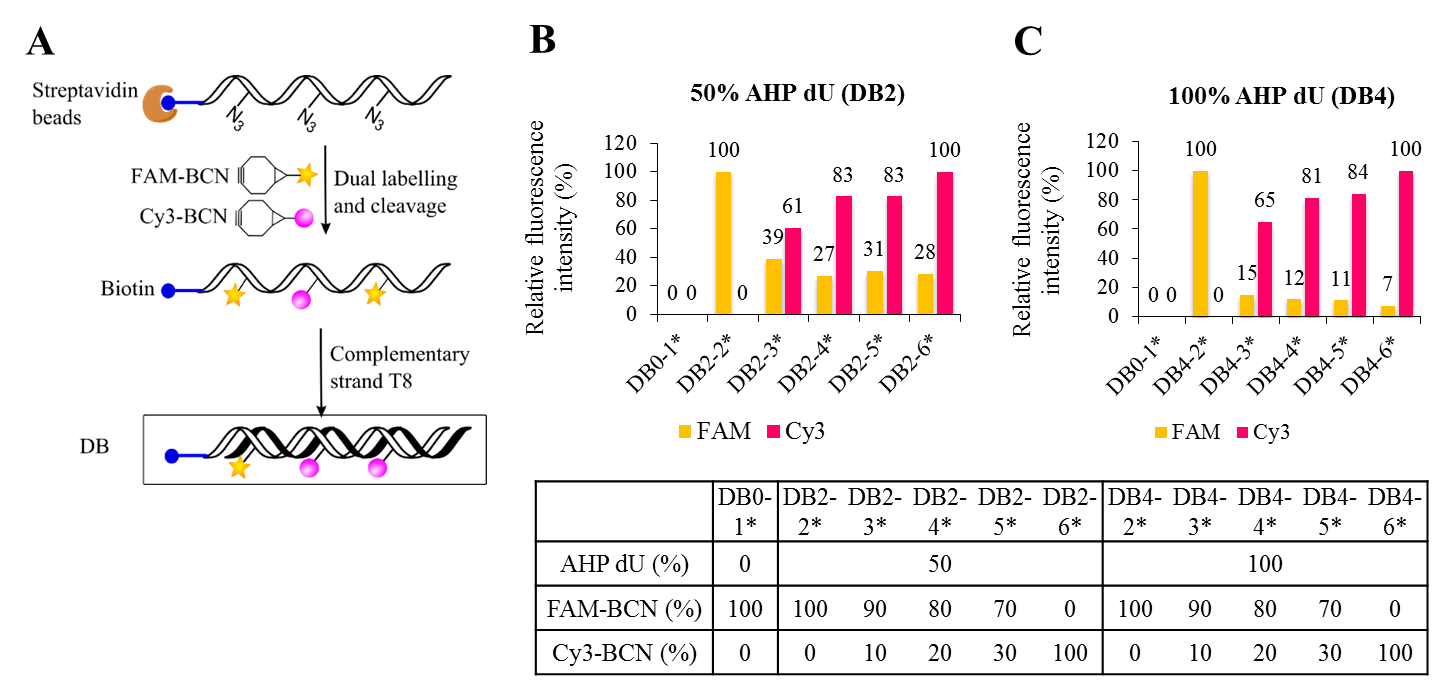


Figure S15. FAM-BCN and Cy3-BCN dual labelling of 50% and 100% AHP-modified single strands (81-mer) from streptavidin magnetic bead separation. **A.** Scheme for dual labelling and re-annealing. **B.** and **C.** Relative fluorescence intensities for the re-annealed duplex products (DB) quantified by gel analyses in FAM and Cy3 channels (quantified using ImageJ). DB0-1^*^, unmodified product mixed with 70% FAM-BCN, negative control; DB2-2^*^/DB4-2^*^ to DB2‑6^*^/DB4‑6^*^, AHP-modified products labelled with different combinations of FAM-BCN (100% to 0%) and Cy3-BCN (0% to 100%) respectively.

#### FAM-BCN and Cy5-BCN dual labelling of ssDNA (T8) on streptavidin magnetic beads


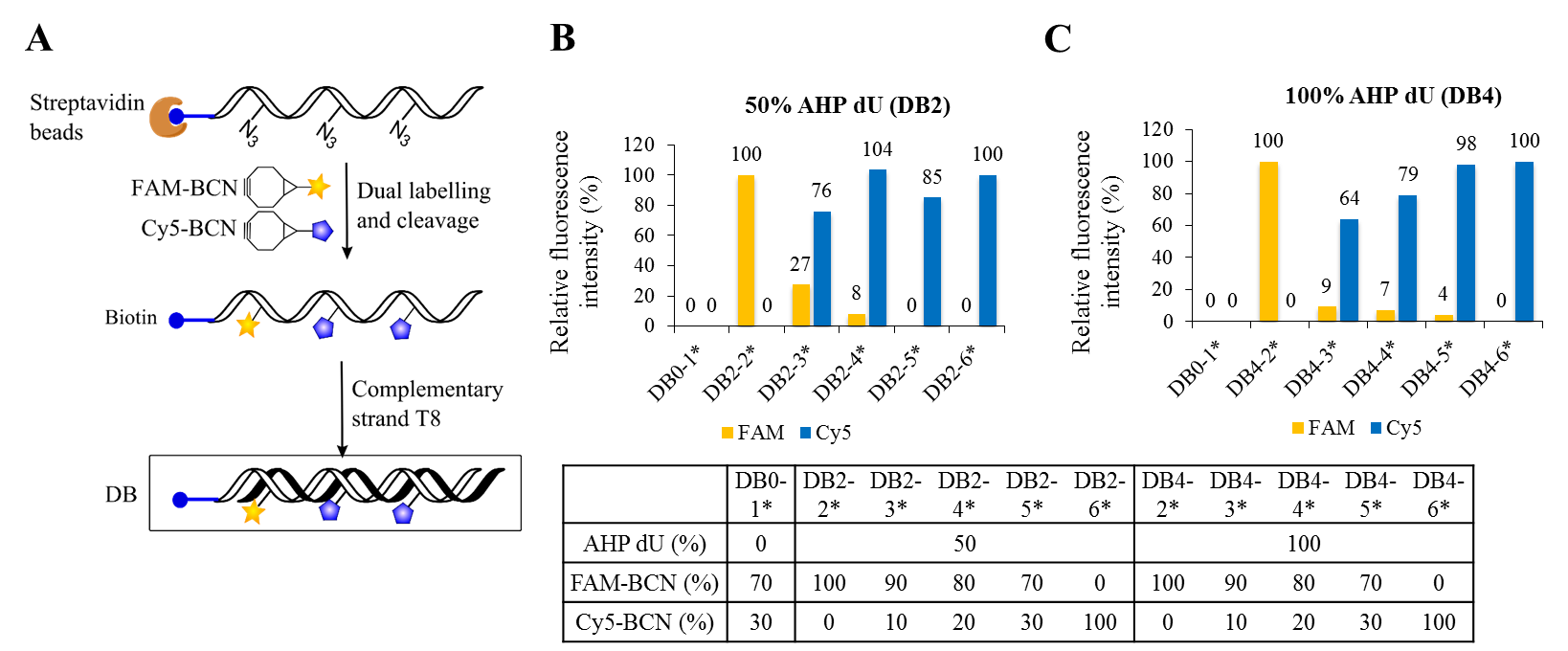


Figure S16. FAM-BCN and Cy5-BCN dual labelling of 50% and 100% AHP-modified single strands (81-mer) from streptavidin magnetic bead separation. **A.** Scheme for dual labelling and re-annealing. **B.** and **C.** Relative fluorescence intensities for the re-annealed duplex products (DB) quantified by gel analyses in FAM and Cy5 channels (quantified using ImageJ). DB0-1^*^, unmodified product with 70% FAM-BCN, negative control; DB2‑2^*^/DB4-2^*^ to DB2‑6^*^/DB4‑6^*^, AHP-modified products labelled with different combinations of FAM-BCN (100% to 0%) and Cy5-BCN (0% to 100%) respectively.

#### Cy3-BCN and Cy5-BCN dual labelling of ssDNA (T8) on streptavidin magnetic beads


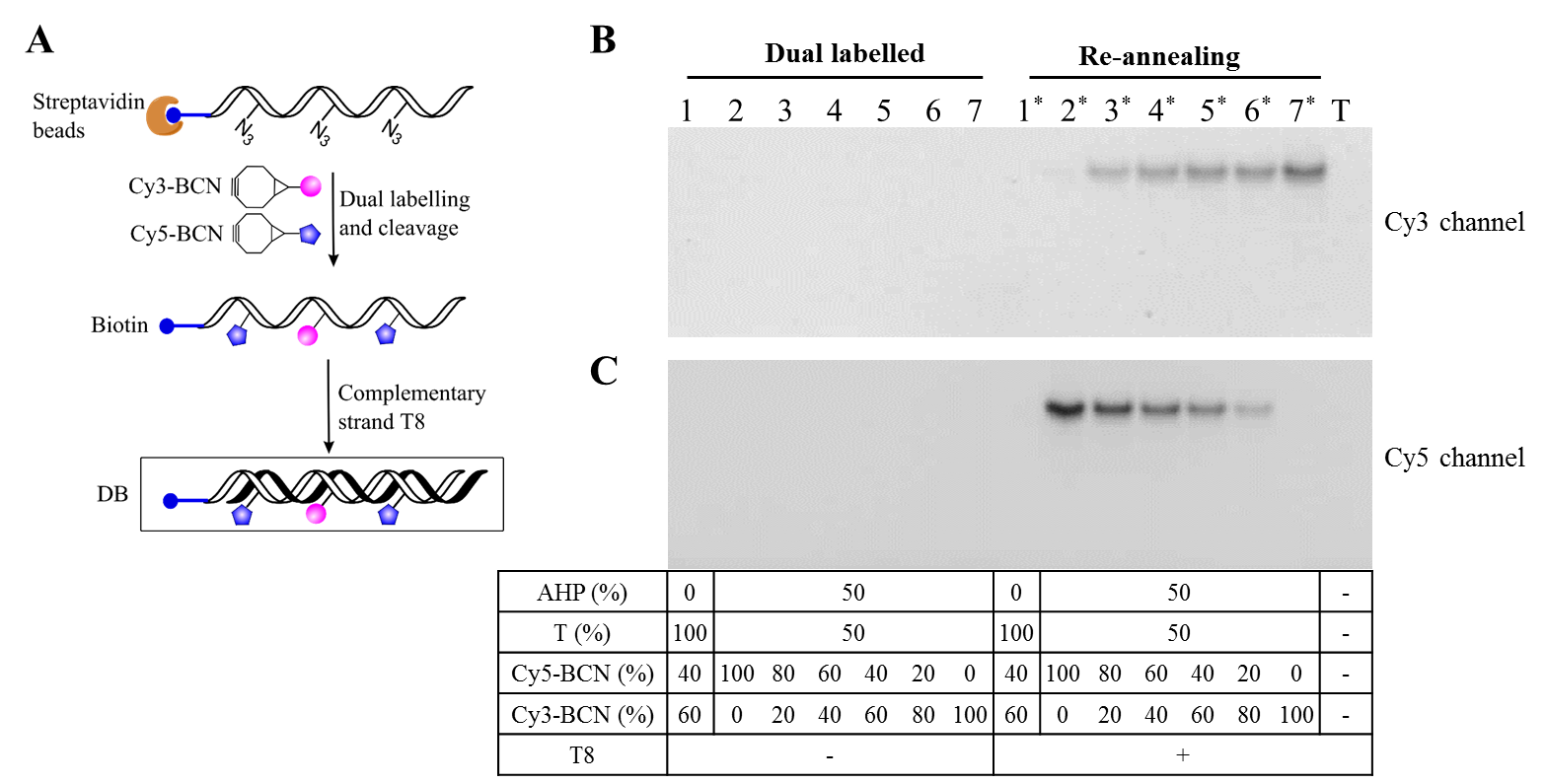


Figure S17. Cy3-BCN and Cy5-BCN dual labelling of 50% AHP-modified single strands (81-mer) from streptavidin magnetic bead separation. **A.** Scheme for dual labelling and re-annealing. **B.** and **C.** 12% native PAGE analysis visualised by Cy3 and Cy5 channels. Lane 1/1^*^, unmodified single strand, negative control; lanes 2/2^*^ to 7/7^*^, 50% AHP‑modified products labelled with different combinations of Cy5-BCN (100% to 0%) and Cy3-BCN (0% to 100%) respectively.


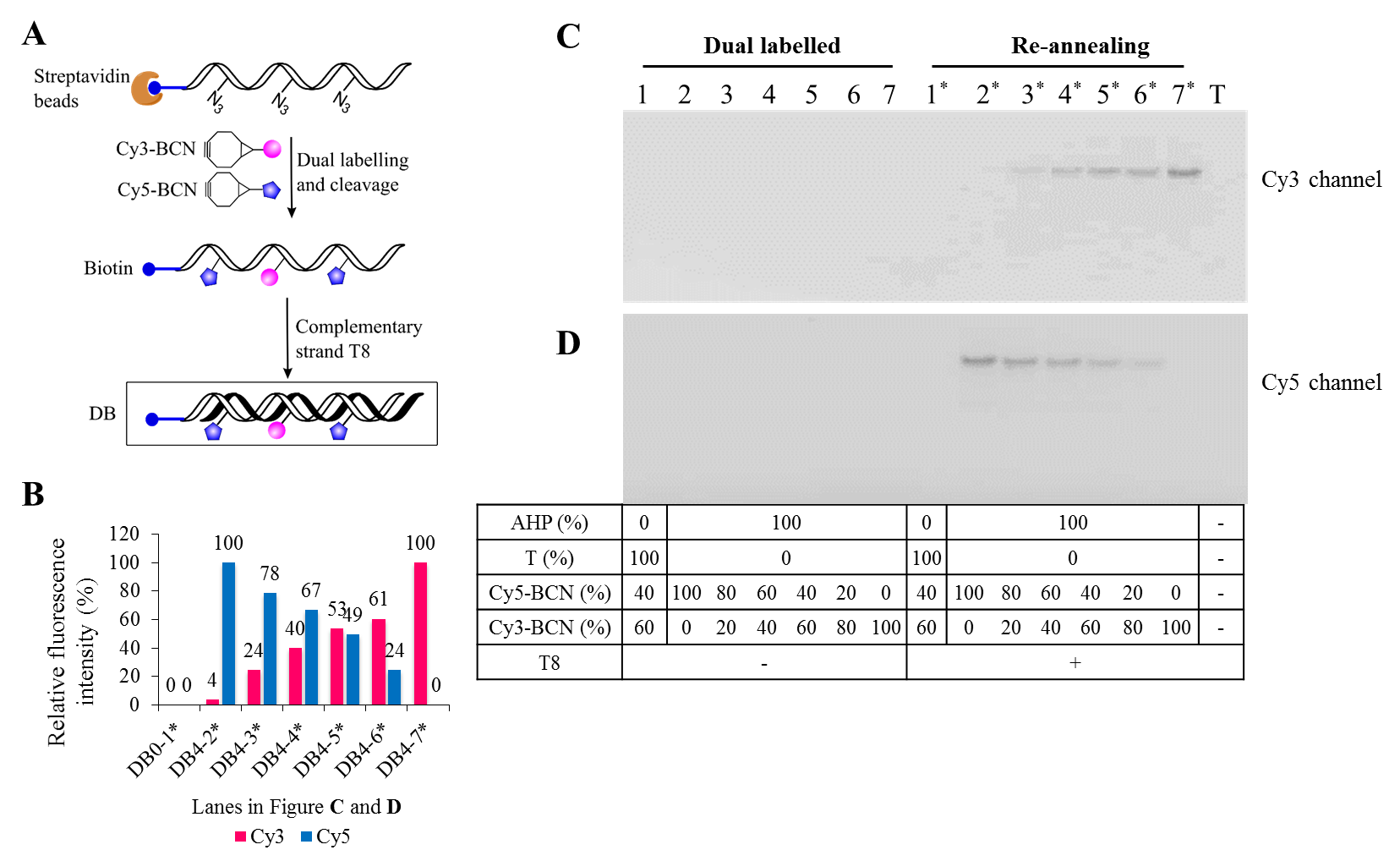


Figure S18. Dual labelling of 100% AHP-modified single strands (81-mer) from streptavidin magnetic bead separation. **A.** Scheme for dual labelling and re-annealing. **B.** Relative fluorescence intensity for re-annealed duplex products quantified by gel analysis in Cy3 and Cy5 channels (lanes 1^*^ to 7^*^ in **C** and **D** quantified using ImageJ). **C.** and **D.** 12% native PAGE analysis visualised in Cy3 channel and Cy5 channel. Lane 1/1^*^, unmodified product, negative control; lanes 2/2^*^ to 7/7^*^, 50% AHP‑modified products labelled with different combinations of Cy5-BCN (100% to 0%) and Cy3-BCN (0% to 100%); lane T, complementary T8 strand.

# References:

1. Ren, X., Gerowska, M., El-Sagheer, A.H. and Brown, T. (2014) Enzymatic incorporation and fluorescent labelling of cyclooctyne-modified deoxyuridine triphosphates in DNA. *Bioorg. Med. Chem.*, **22**, 4384-4390.

2. Jäger, S., Rasched, G., Kornreich-Leshem, H., Engeser, M., Thum, O. and Famulok, M. (2005) A versatile toolbox for variable DNA functionalization at high density. *J. Am. Chem. Soc.* , **127**, 15071-15082.

3. Lampkins, A.J., O’Neil, E.J. and Smith, B.D. (2008) Bio-orthogonal phosphatidylserine conjugates for delivery and imaging applications. *J. Org. Chem.* , **73**, 6053-6058.

4. Kvach, M.V., Ustinov, A.V., Stepanova, I.A., Malakhov, A.D., Skorobogatyi, M.V., Shmanai, V.V. and Korshun, V.A. (2008) A convenient synthesis of cyanine dyes: reagents for the labeling of biomolecules. *Eur. J. Org. Chem.* , **2008**, 2107-2117.

5. Ren, X., El-Sagheer, A.H. and Brown, T. (2015) Azide and trans-cyclooctene dUTPs: incorporation into DNA probes and fluorescent click-labelling. *Analyst*, **140**, 2671-2678.

6. Pierce, K.E., Sanchez, J.A., Rice, J.E. and Wangh, L.J. (2005) Linear-after-the-exponential (LATE)-PCR: primer design criteria for high yields of specific single-stranded DNA and improved real-time detection. *Proc. Natl. Acad. Sci. U.S.A.*, **102**, 8609-8614.

7. Sanchez, J.A., Pierce, K.E., Rice, J.E. and Wangh, L.J. (2004) Linear-after-the-exponential (LATE)-PCR: an advanced method of asymmetric PCR and its uses in quantitative real-time analysis. *Proc. Natl. Acad. Sci. U.S.A.*, **101**, 1933-1938.
